# Supplementary material for: Rapid and on-site wireless immunoassay of respiratory virus aerosols via hydrogel-modulated resonators
Source: Nat Commun. 2024 May 13;15:4035. doi: 10.1038/s41467-024-48294-1 (PMC11091083; doi:10.1038/s41467-024-48294-1)
Supplement: Supplementary file 1 — Supplementary Information [file 41467_2024_48294_MOESM1_ESM.pdf]

# Supplementary information

for

# Rapid and on-site wireless immunoassay of respiratory virus aerosols via hydrogel-modulated resonators

Xin Li,[1,2,6] Rujing Sun,[1,3,6] Jingying Pan,[1,4] Zhenghan Shi,[1] Zijian An,[1] Chaobo Dai,[1] Jingjiang Lv,[1] Guang Liu,[1] Hao Liang,[3] Jun Liu,[1,2] Yanli Lu,[1,5] Fenni Zhang,[1] Qingjun Liu[1,2*]

[1]Biosensor National Special Laboratory, Key Laboratory for Biomedical Engineering of Education Ministry, Department of Biomedical Engineering, Zhejiang University, Hangzhou, 310027, China

[2]Taizhou Key Laboratory of Medical Devices and Advanced Materials, Research Institute of Zhejiang University-Taizhou, Taizhou 318000, China

[3]Guangxi Key Laboratory of AIDS Prevention and Treatment, School of Public Health, Biosafety III Laboratory, Life Science Institute, Guangxi Medical University, Nanning 530021, Guangxi, China

[4]School of Medicine, Zhejiang University, Hangzhou, 310027, China

[5]Intelligent Perception Research Institute, Zhejiang Lab, Hangzhou 311100, China

[6]These authors contributed equally: Xin Li, Rujing Sun

Email: qjliu@zju.edu.cn

## Contents

**Supplementary Fig. 1** Synthesis of vinyl-antigen and AuNPs-Ab for immuno-responsive hydrogel preparation.
**Supplementary Fig. 2** Measuring of the dissociation equilibrium constant (KD) of antigen-antibody by SPR.
**Supplementary Fig. 3** Shear storage modulus of hydrogels before/after competitive immuno binding.
**Supplementary Fig. 4** Characterization of AuNPs and AuNPs-Ab before and after antigen binding.
**Supplementary Fig. 5** Characterization of the swelling ratio and average micropore size of the hydrogels.
**Supplementary Fig. 6** Fabrication of split ring resonators and ImmHR sensor assembly.
**Supplementary Fig. 7** Simulation and experimental tests of differently configured resonator sensors.
**Supplementary Fig. 8** Paralleled IR mediated RF readout network.
**Supplementary Fig. 9** Characterization of the nanopore induced by immobilized AuNPs.
**Supplementary Fig. 10** Response of the RGB parameters in colorimetric assays.
**Supplementary Fig. 11** Kinetics of the hydrogel colorimetric response in SARS-CoV-2 NP spiked solution.
**Supplementary Fig. 12** Linear fitting of the hydrogel colorimetric response in SARS-CoV-2 NP spiked solution and aerosol.
**Supplementary Fig. 13** Detection of SARS-CoV-2 NP in different liquid volumes.
**Supplementary Fig. 14** Schematic of the aerosol generation and testing.
**Supplementary Fig. 15** Influence of sensor displacement without IR coils.
**Supplementary Fig. 16** Configuration of the wireless immunoassay.
**Supplementary Fig. 17** Influence of bending radii on sensor readout.
**Supplementary Fig. 18** $F_{res}$ distribution and reproducibility of as-prepared ImmHR sensors.
**Supplementary Fig. 19** Influence of hydrogel thickness on aerosol detection.
**Supplementary Fig. 20** Comparison of the hydrogel network crosslinked by AuNP-Ab and antibody.
**Supplementary Fig. 21** Unnormalized response curves of the ImmHR sensors for SARS-CoV-2 NP, H1N1 HA, and RSV FP aerosol detection.
**Supplementary Fig. 22** Calibration plot of the SARS-CoV-2 and H1N1 sensors at 30 min.
**Supplementary Fig. 23** Rapid aerosol detection capability of the SARS-CoV-2 and H1N1 sensors.
**Supplementary Fig. 24** Air permeability of face masks with and without the wireless immunoassay devices.
**Supplementary Fig. 25** Influence of preservation humidity on hydrogel dehydration.
**Supplementary Fig. 26** Stability of the ImmHR sensors in two weeks.
**Supplementary Fig. 27** Original response of clinical samples from patients with H1N1.
**Supplementary Fig. 28** Original response of samples from healthy people.
**Supplementary Fig. 29** Original response of clinical samples from suspected positive and fever yet H1N1 negative patients.
**Supplementary Fig. 30** Commercial LFA strips for H1N1 detection.
**Supplementary Fig. 31** Comparison of nucleic acid testing and immunoassays for virus detection.
**Supplementary Table 1** Ct values and ImmHR sensor response of clinical samples.
**Supplementary Table 2** Comparison of COVID-19 detection methods and this work.
**Supplementary references**

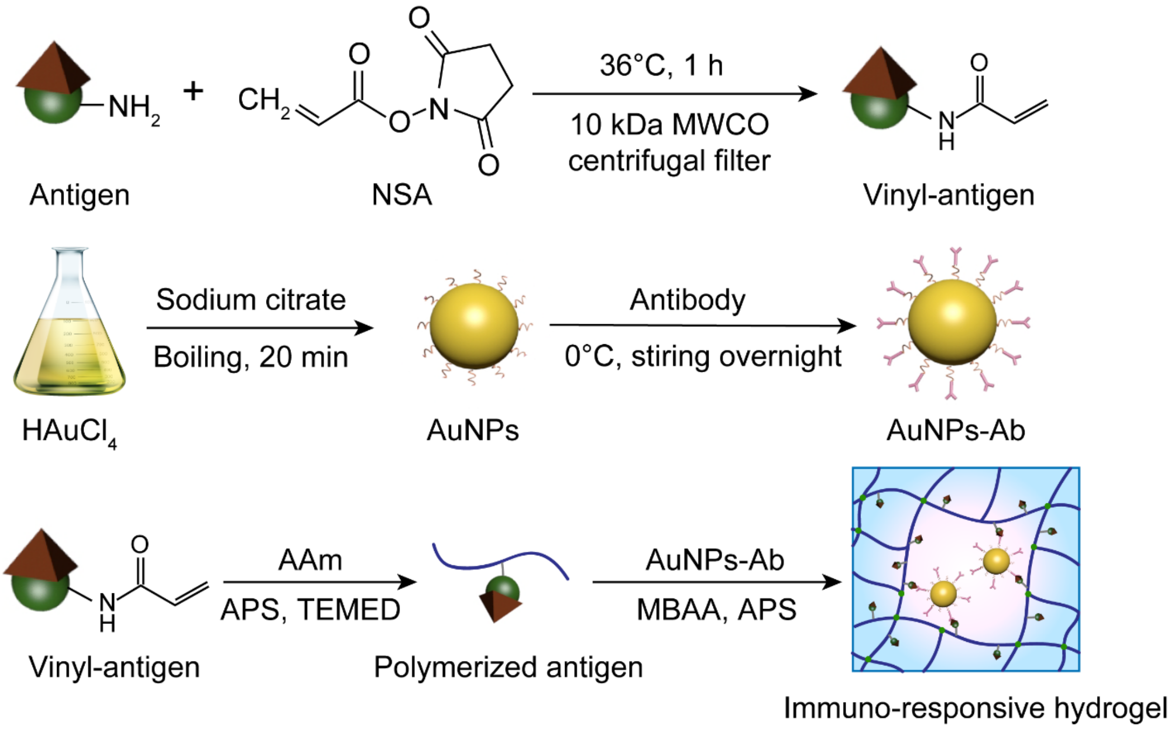


**Supplementary Fig. 1 Synthesis of vinyl-antigen and AuNPs-Ab for immuno-responsive hydrogel preparation.** Details of the synthesis process is illustrated in Methods.

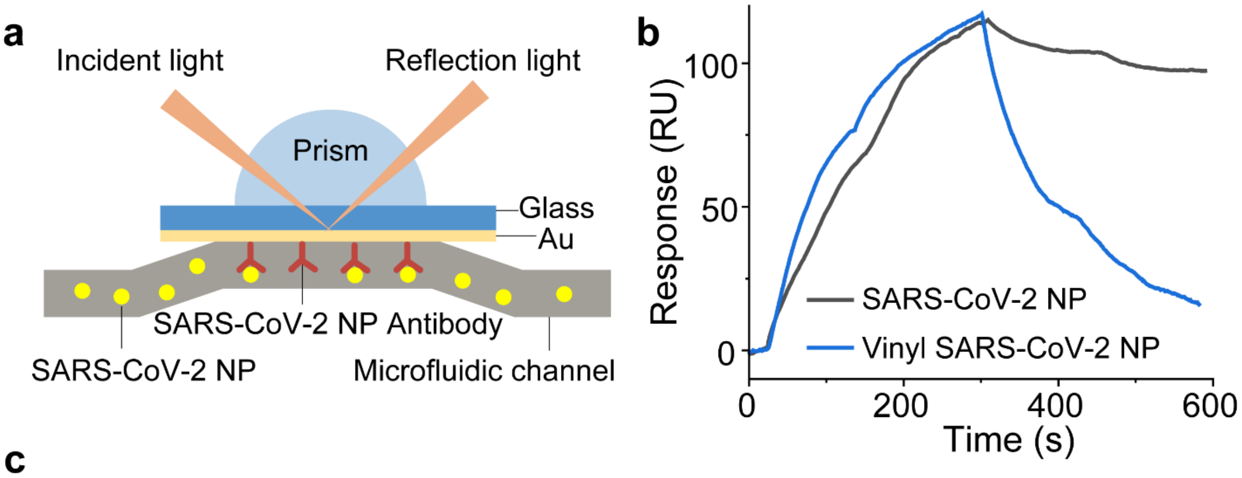


c

| Fitting model | | NP | Vinyl-NP |
|---|---|---|---|
| $R=\frac{k_a C_0 R_{max}}{k_a C_0+k_d}\left[1-e^{-(k_a C_0+k_d)t}\right]$ | $k_a$ | $3\times10^{5}$ | $1.8\times10^{5}$ |
| | $k_d$ | $7\times10^{-4}$ | $7.7\times10^{-3}$ |
| | $k_D=k_d/k_a$ | $2.4\times10^{-9}$ | $4.2\times10^{-8}$ |

**Supplementary Fig. 2 Measuring of the dissociation equilibrium constant ($K_D$) of antigen-antibody by SPR.**

(a) Principle of the SPR measurement to determine binding strength between SARS-CoV-2 NP and antibody. (b) Dynamic SPR intensity induced by SARS-CoV-2 NP (pristine antigen) and vinyl-SARS-CoV-2 NP (vinyl-antigen) binding to antibody modified gold chips. (c) Fitting model of (b), and resolved $k_D$ values of pristine antigen and vinyl-antigen.

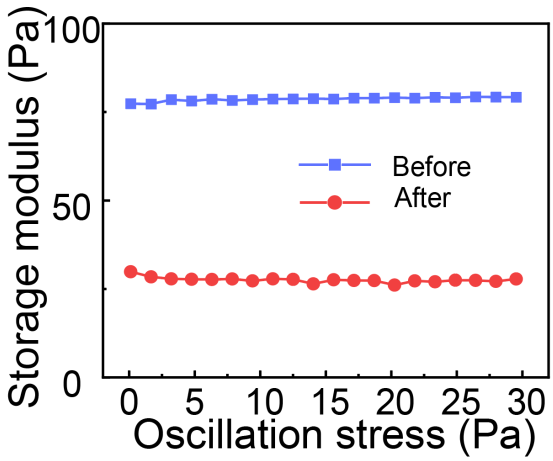


**Supplementary Fig. 3 Shear storage modulus of hydrogels before/after competitive immuno binding.**

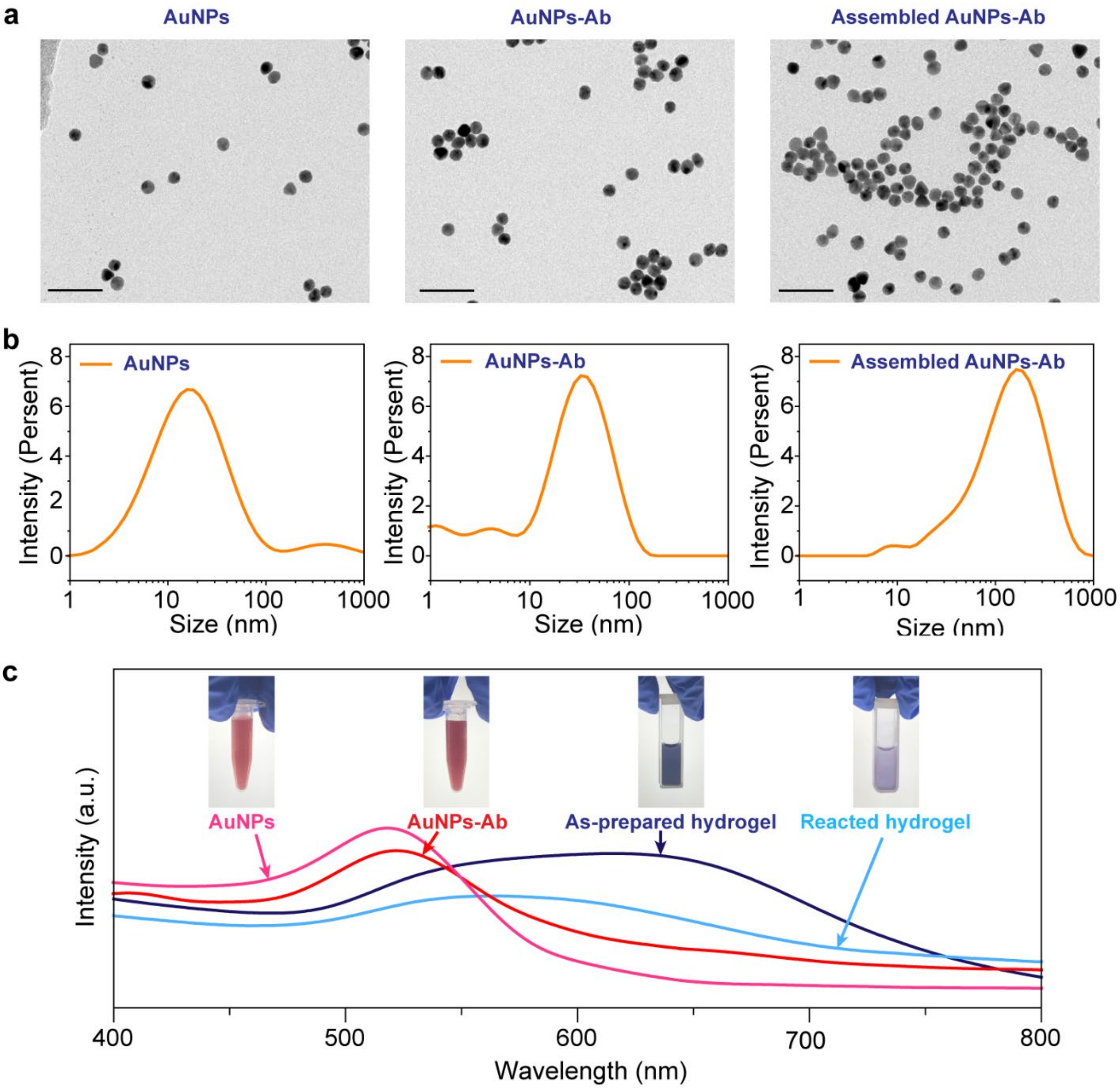


**Supplementary Fig. 4 Characterization of AuNPs and AuNPs-Ab before and after antigen binding.**

(a) TEM images of AuNPs, AuNPs-Ab, and AuNPs-Ab assembled by 1 ng/mL antigen in solution, respectively. (b) Size distribution of AuNPs, AuNPs-Ab, and AuNPs-Ab aggregated by 1 ng/mL antigen in solution, respectively. (c) UV-vis spectra and photos revealing the color change of AuNPs during the hydrogel formation and detection. Results from (c) indicates that AuNPs underwent moderate aggregation during hydrogel formation, as the color turns from red to blue (corresponds to AuNPs aggregates of hundreds of nanometer[1]). Further reaction with pristine antigen leads to severe AuNPs aggregation that cannot disperse in the hydrogel, and the blue fades out. All the experiments are repeated three times with similar results.

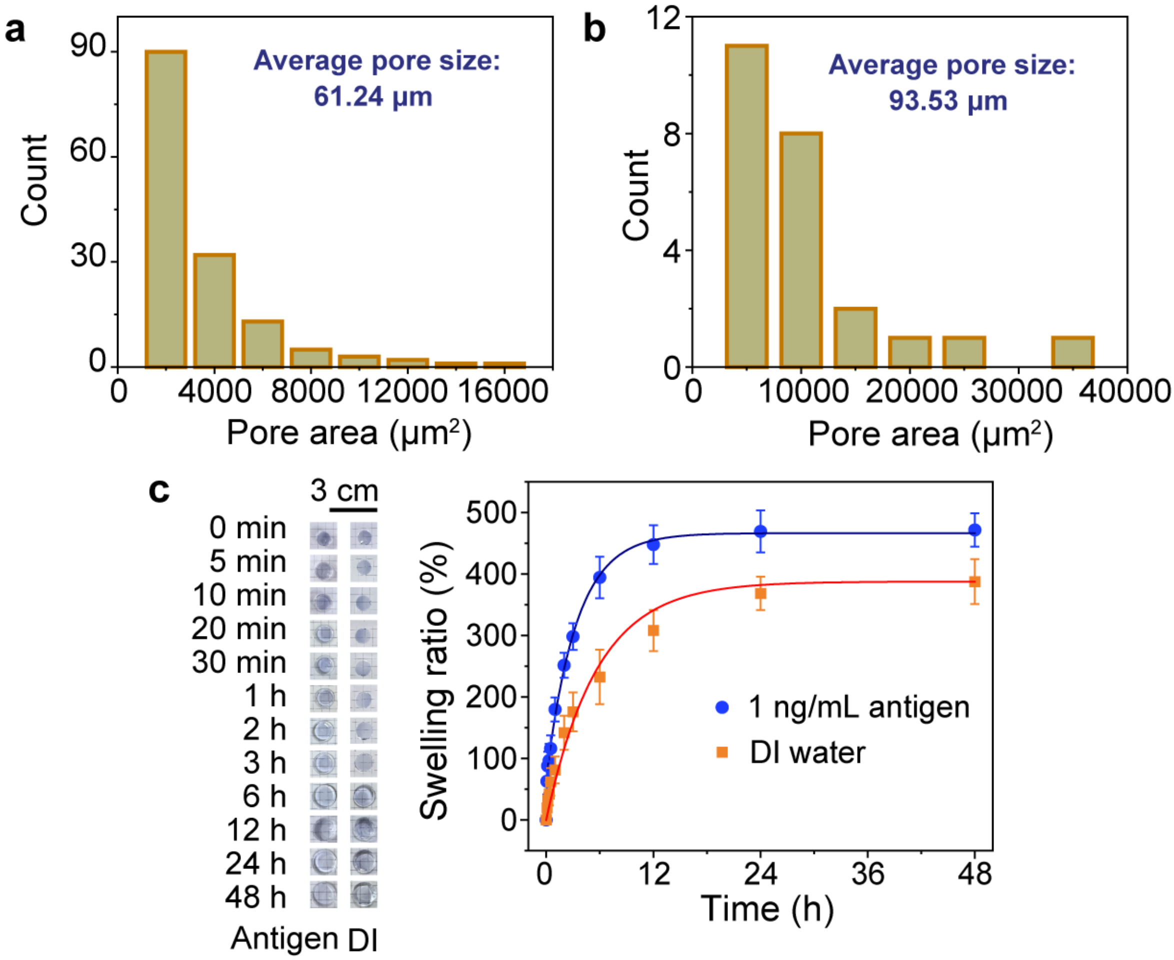


**Supplementary Fig. 5 Characterization of the swelling ratio and average micropore size of the hydrogels.**

(a) Distribution of micropores in the as-prepared hydrogel. (b) Distribution of micropores in hydrogel reacted with 1 ng/mL antigen for 3 h. The experiments are repeated three times with similar results. (c) Swelling ratio of the hydrogel in DI water and 1 ng/mL antigen spiked solution. Left panel shows the optical images of swelled hydrogels. Error bars are displayed as mean ± σ (n=3 independent experiments).

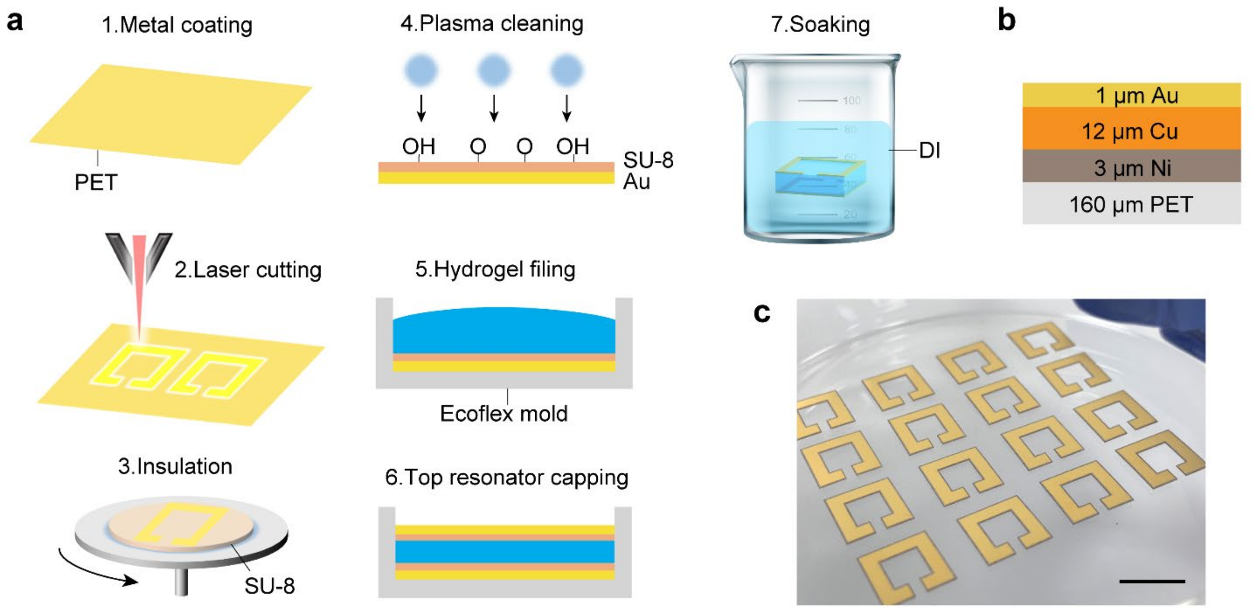


**Supplementary Fig. 6 Fabrication of split ring resonators and ImmHR sensor assembly.** (a) Fabrication process of the ImmHR sensors. (b) Thickness of metal foils of resonators. (c) Photo of laser-cut resonators, which is suitable for scalable manufacturing. The scale bar is 1 cm.

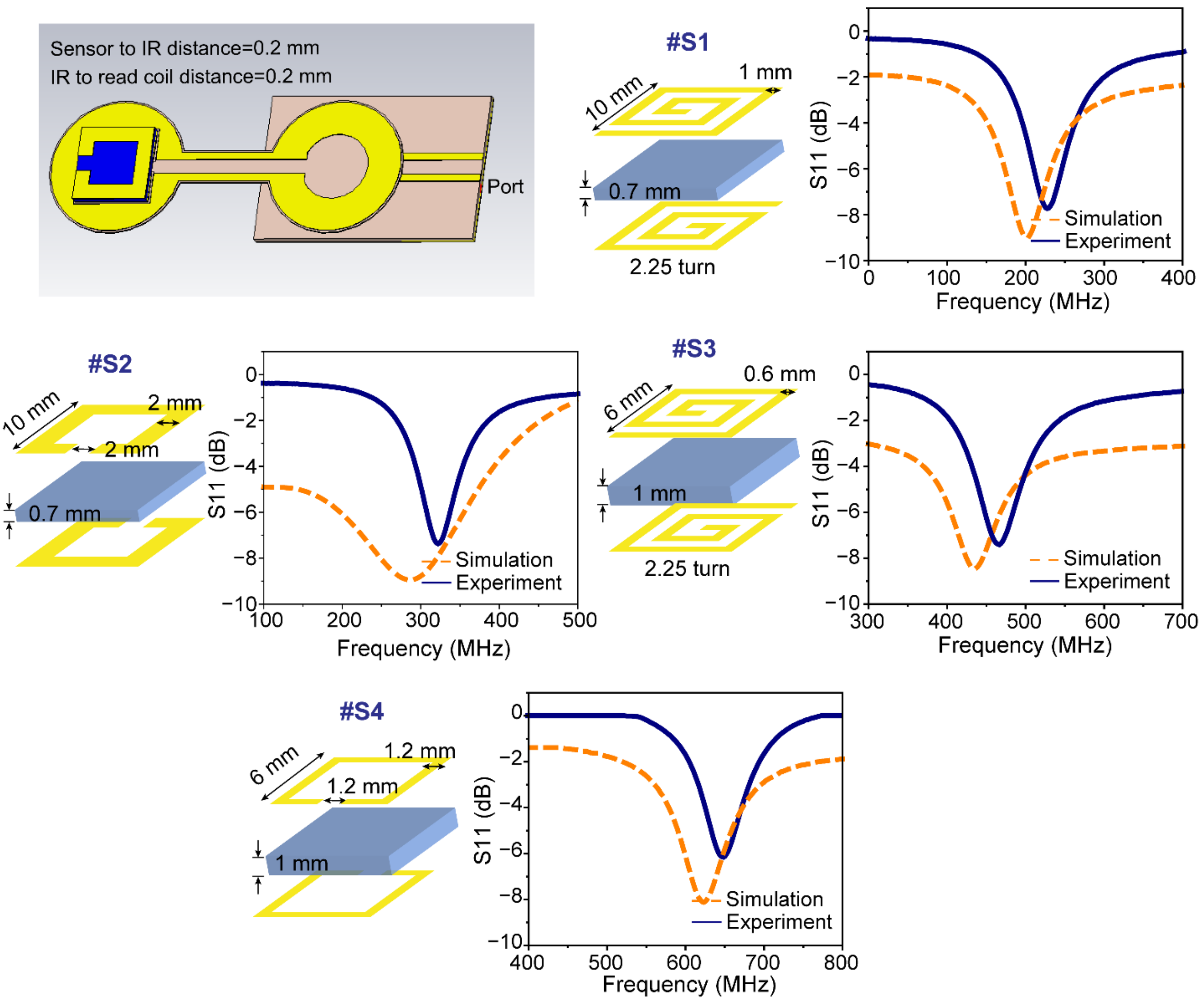


**Supplementary Fig. 7 Simulation and experimental tests of differently configured resonator sensors.** All the resonators are fabricated with the same protocols. The finite-difference time-domain is adopted for the electromagnetic simulation.[2]

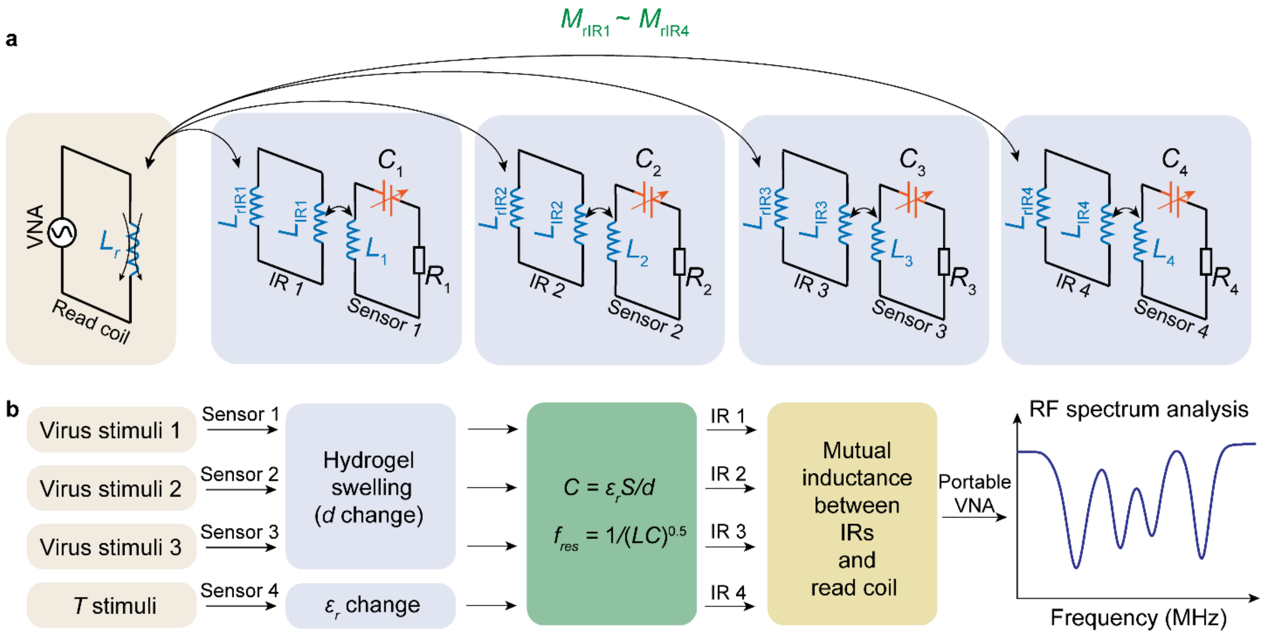


**Supplementary Fig. 8 Paralleled IR mediated RF readout network.**

(a) Schematic illustration of paralleled RF readout network mediated by IRs. (b) Workflow of signal transduction for RF spectrum analysis.

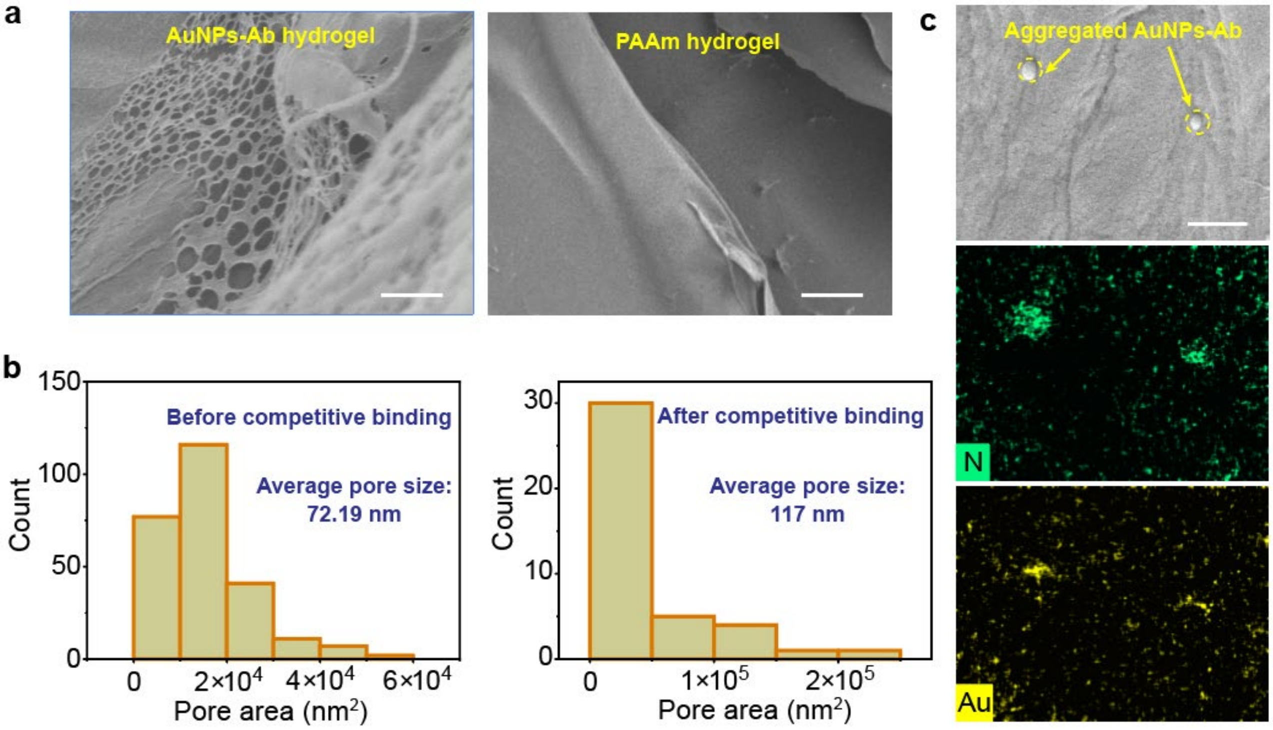


**Supplementary Fig. 9 Characterization of the nanopore induced by immobilized AuNPs.** (a) Nanoscale morphologies of AuNPs-Ab hydrogel and PAAm hydrogel. Scale bar: 500 nm. (b) Size distribution of the nanopores of AuNPs-Ab hydrogel before/after 1 ng/mL antigen stimuli. (c) SEM images and element mapping of the aggregated AuNPs-Ab. Scale bar: 1 μm. The focused distribution of Au indicates the existence of AuNPs, while the focused N distribution is attributed to the encapsulation of antigen/antibody proteins. All the experiments are repeated three times with similar results.

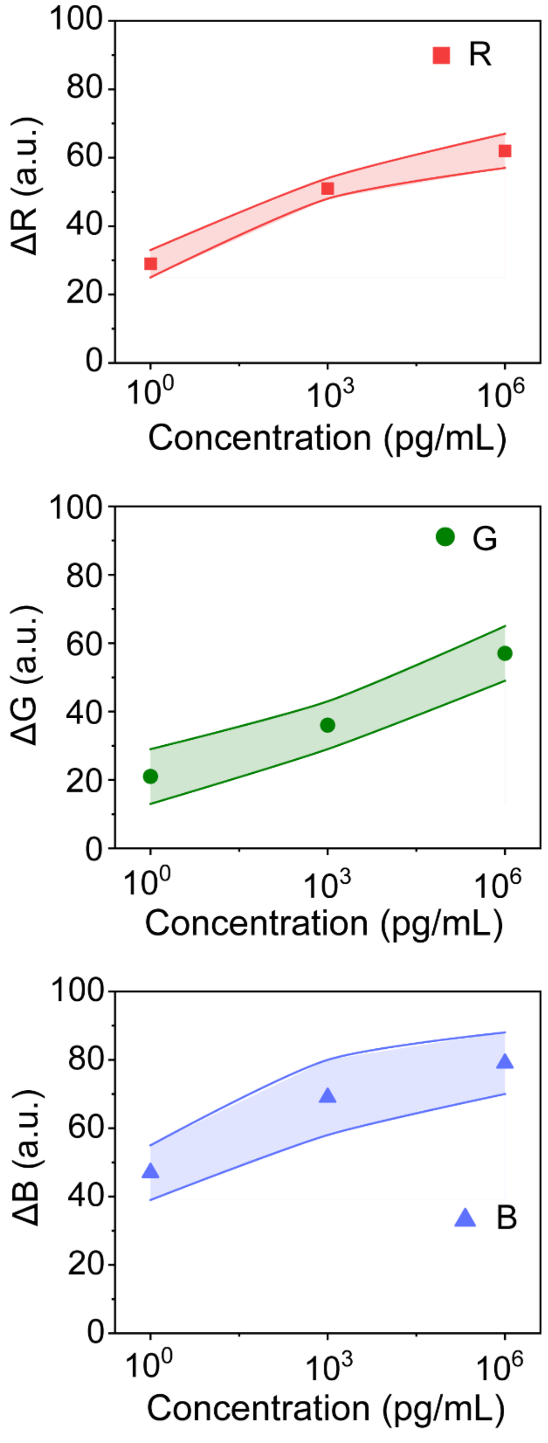


**Supplementary Fig. 10 Response of the RGB parameters in colorimetric assays.** The color strips represent the standard deviation (n=3 independent tests). The relative standard deviation (RSD) are 5.8%~13.7%, 14.0~38.1%, and 11.3~17.0% for R, G, B, respectively.

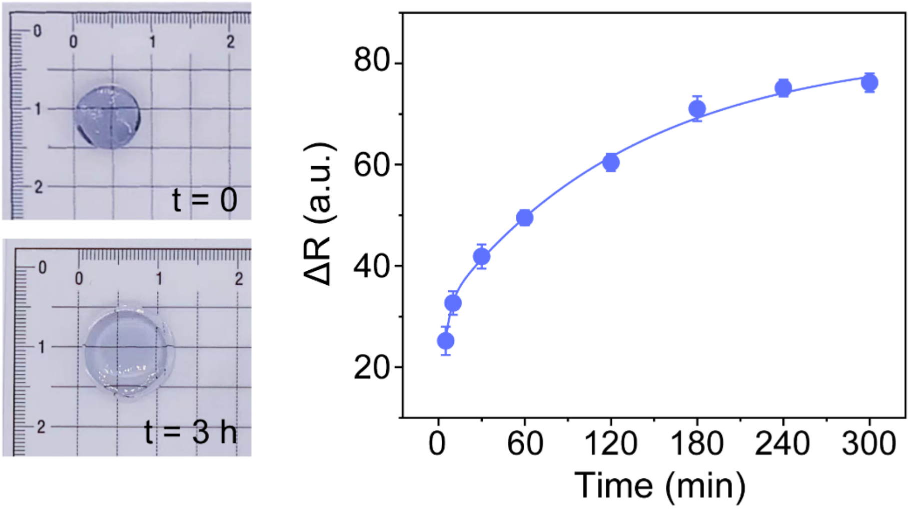


**Supplementary Fig. 11 Kinetics of the hydrogel colorimetric response in SARS-CoV-2 NP spiked solution.** Left: Photos of hydrogels on filming ruler to show the swelling and color change. Right: Equilibrium colorimetric response of the hydrogels in 1 ng/mL antigen for 3 h. Error bars are displayed as mean ± σ (n=3 independent experiments).

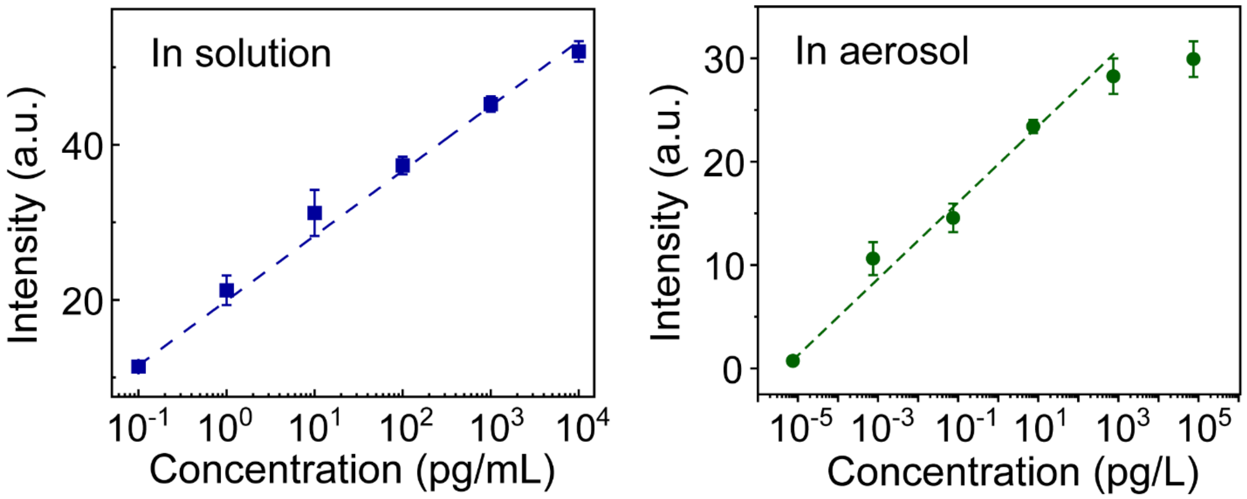


**Supplementary Fig. 12 Linear fitting of the hydrogel colorimetric response in SARS-CoV-2 NP spiked solution and aerosol.** The data is acquired after 90 min incubation. Error bars are displayed as mean ± σ (n=3 independent experiments).

.

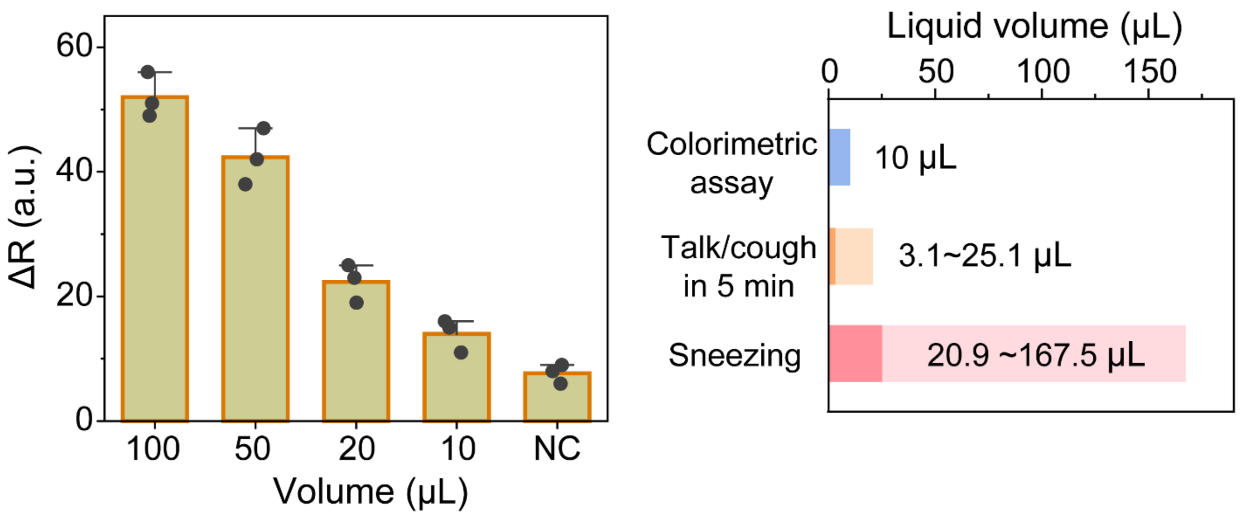


**Supplementary Fig. 13 Detection of SARS-CoV-2 NP in different liquid volumes.** Left: Colorimetric response in 100, 50, 20, and 10 μL of 1 ng/mL antigen, compared with 10 μL NC. Right: Exhaled liquid volume from talk/cough and sneezing,[3] compared with that needed for colorimetric assay. Error bars are displayed as mean ± σ (n=3 independent experiments).

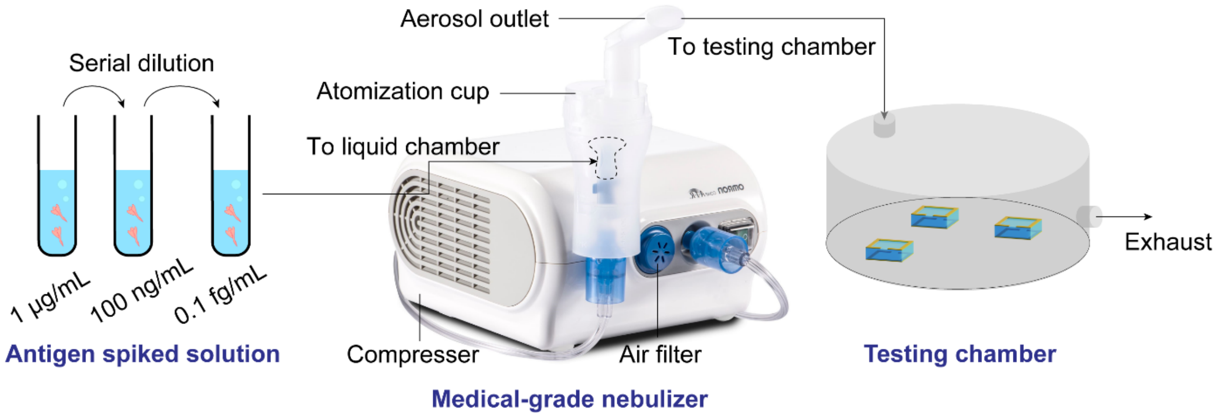


**Supplementary Fig. 14 Schematic of the aerosol generation and testing.** The antigens were spiked in 1× PBS to obtain 1 μg/mL stock, which was diluted into desirable concentration. For aerosol generation, about 3~4 mL spiked solution was added into the liquid chamber of the atomization cup, which was processed into aerosols with particle size below 5 μm. The aerosols were delivered from the medical-grade nebulizer to the testing chamber via silicone tubes. The whole aerosol generation system was washed by DI water three times before switching to the next test.

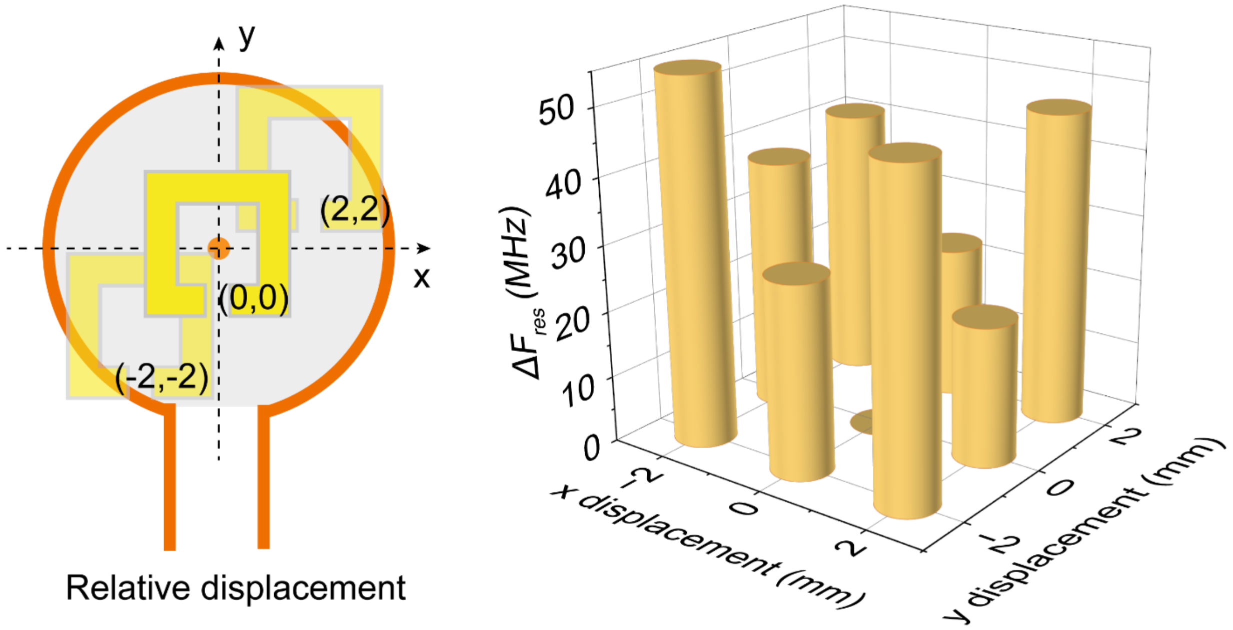


**Supplementary Fig. 15 Influence of sensor displacement without IR coils.** Left: Illustration of the origin point and direction of read coil displacement. Right: Resonant frequency shift upon read coil displacement.

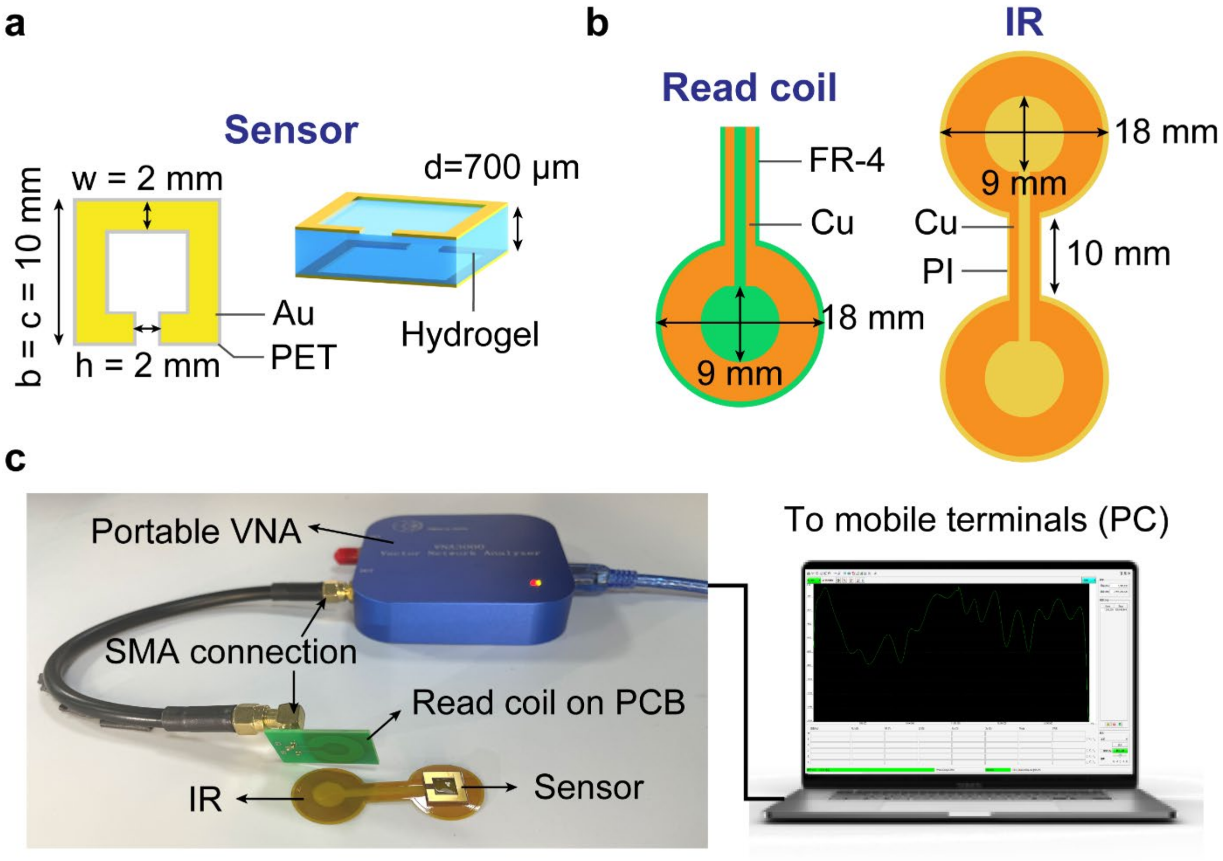


**Supplementary Fig. 16 Configuration of the wireless immunoassay.**

(a) A typical configuration of ImmHR sensor. (b) The typical configuration of IR and read coils.(c) A photo illustrates the wireless detection with ImmHR sensor, IR, read coil, and a portable VNA.

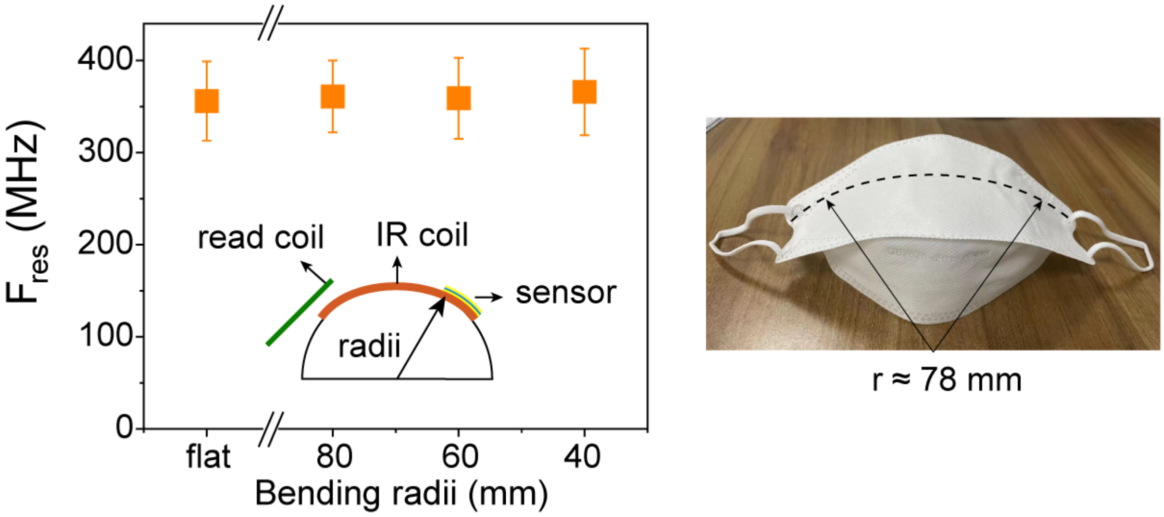


**Supplementary Fig. 17 Influence of bending radii on sensor readout.** Left: Resonant frequency of the ImmHR sensor on IR with bending radii of 40, 60, 80 mm, compared with flat status. Right: The bending radii of a curved face mask. Error bars are displayed as mean ± σ (n=3 independent experiments).

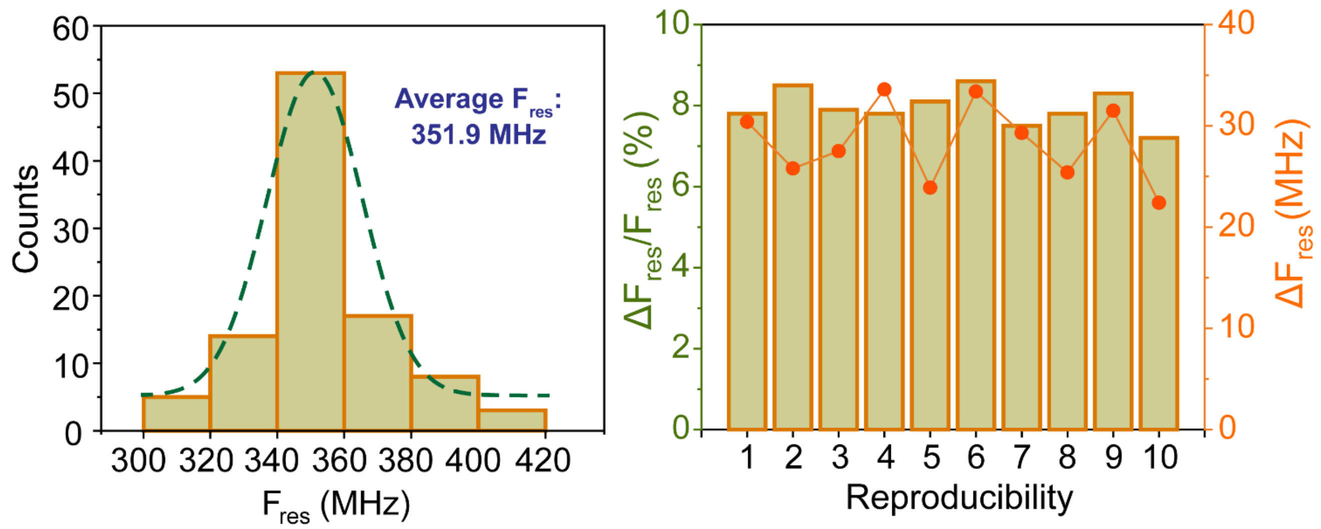


**Supplementary Fig. 18 $F_{res}$ distribution and reproducibility of as-prepared ImmHR sensors.** Left: $F_{res}$ distribution of 100 chips of as-prepared ImmHR sensors. Right: Reproducibility of the ImmHR sensors from 10 batches, compared by normalized response and resonant frequency shift. The responses were tested in 1 ng/mL antigen. The experiments are repeated three times with similar results.

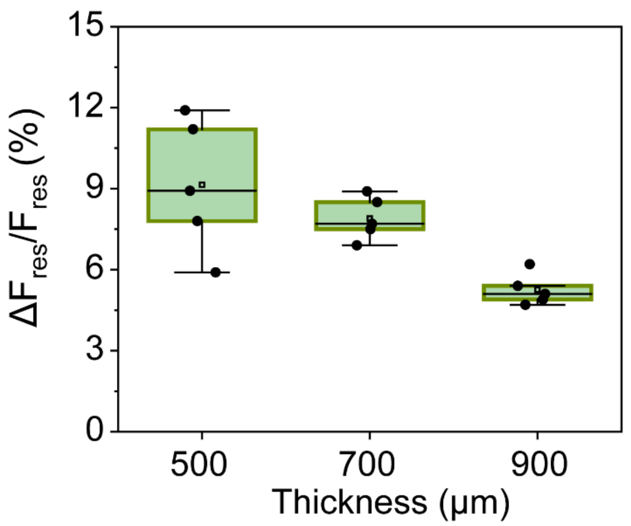


**Supplementary Fig. 19 Influence of hydrogel thickness on aerosol detection.** The responses were tested in 1 ng/mL antigen (n=5 independent tests). The thickness was controlled by the volume of hydrogel precursor. Central lines indicate the median value, box limits represent the upper and lower quartiles, and whiskers indicate 1.5× the interquartile range above and below the upper and lower quartiles, respectively.

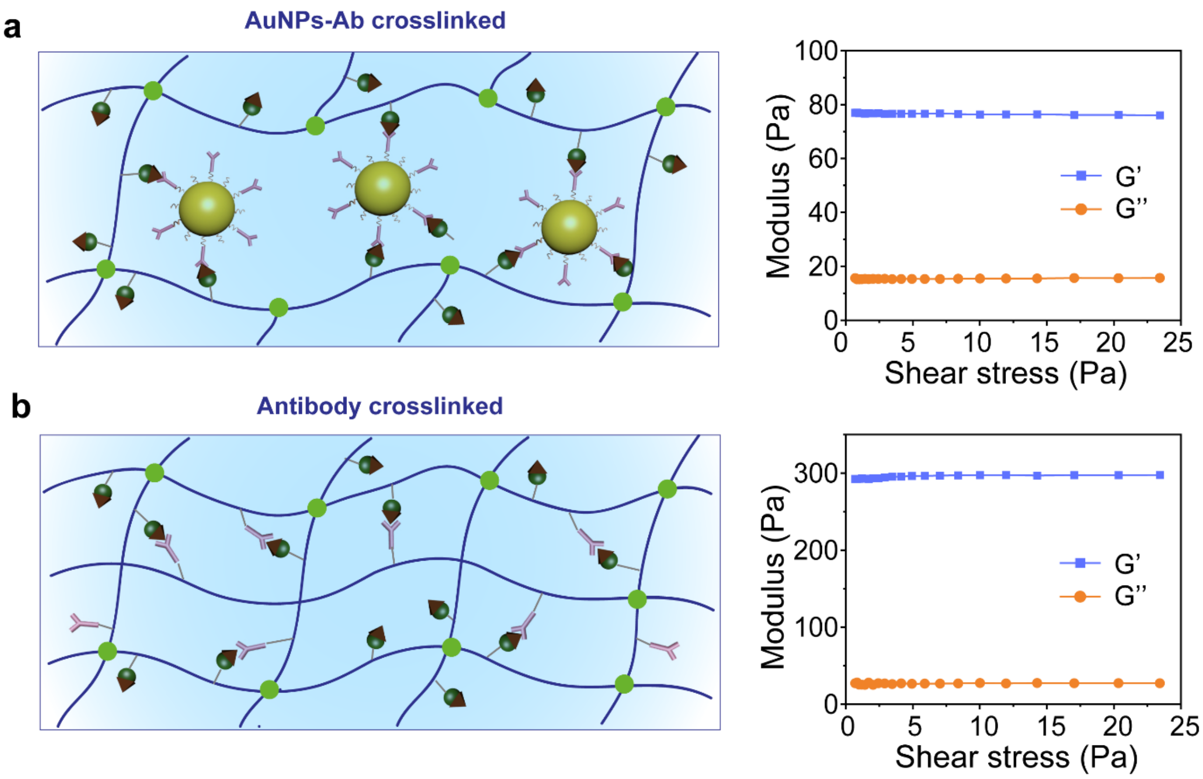


**Supplementary Fig. 20 Comparison of the hydrogel network crosslinked by AuNP-Ab and solely antibody.**

(a) Schematic illustration of the hydrogel network crosslinked by AuNPs-Ab and its modulus. (b) Schematic illustration of the hydrogel network crosslinked by antibody and its modulus. G', storage modulus. G'', loss modulus. The G' is higher than G'', which indicates that both hydrogels are elastic solid. Nevertheless, the G' of AuNPs-Ab crosslinked hydrogel is much lower than that of antibody-crosslinked ones, indicating AuNPs-Ab crosslinked hydrogel is easier to undergo elastic deformation, in our case, more responsive to antigen stimuli and produce more sensitive swelling. The experiments are repeated three times with similar results.

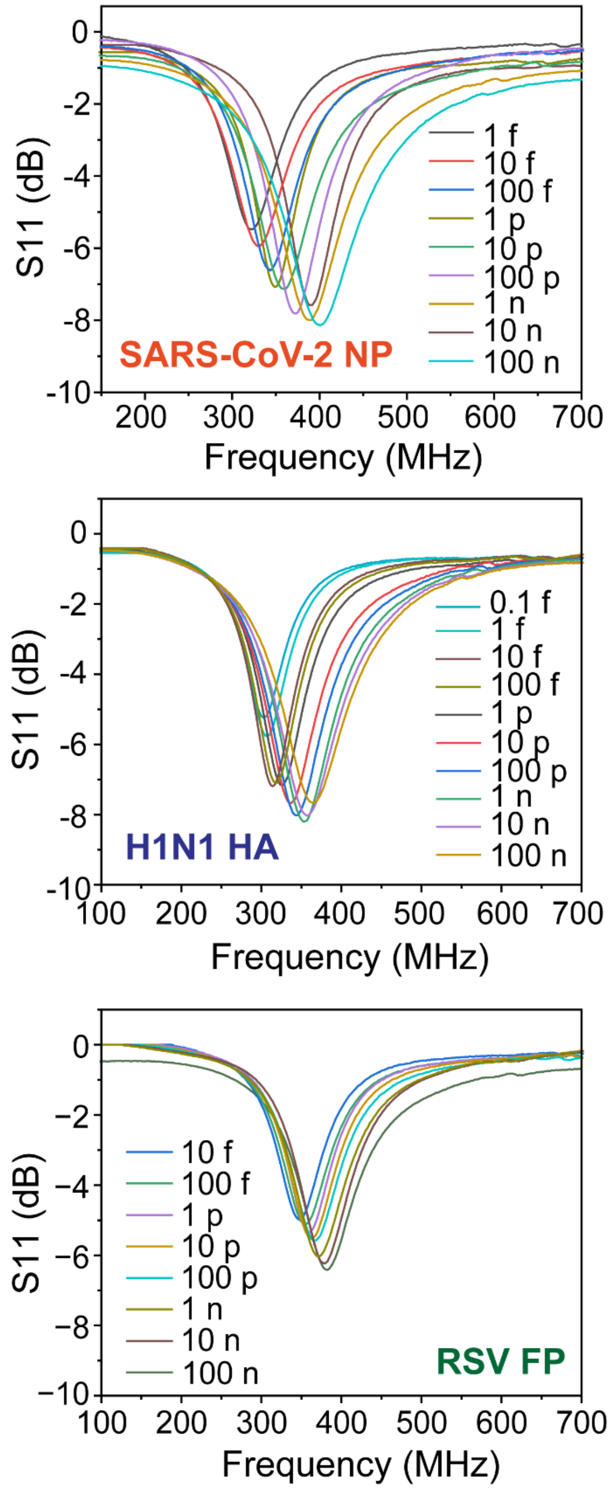


**Supplementary Fig. 21 Unnormalized response curves of the ImmHR sensors for SARS-CoV-2 NP, H1N1 HA, and RSV FP aerosol detection.** The concentrations represent the spiked solution for aerosol generation, which has a conversion factor of 0.0714 from pg/mL in solution to pg/L in aerosol, as detailed in Methods. The responses were acquired at 10 min.

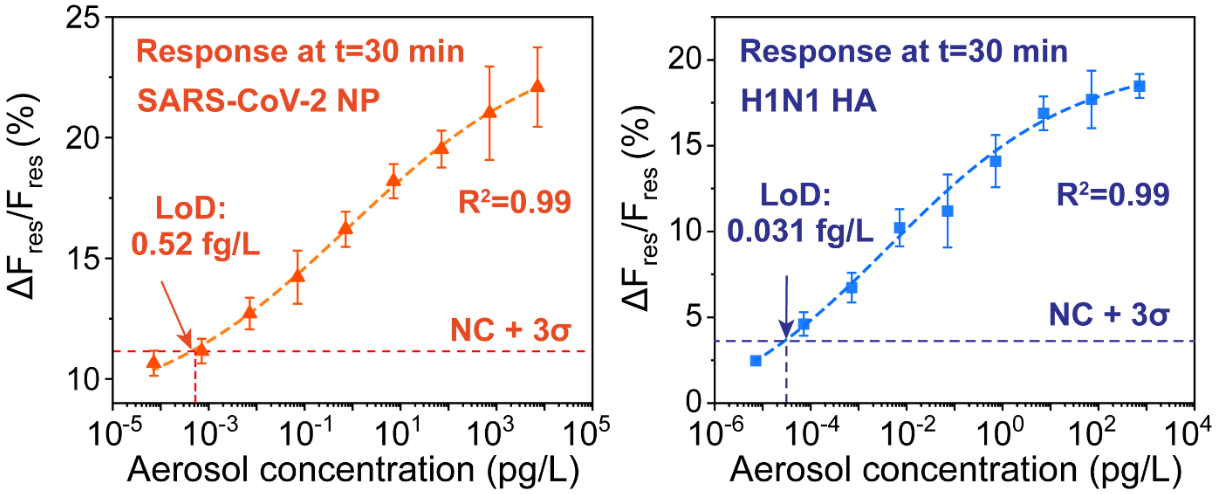


**Supplementary Fig. 22 Calibration plot of the SARS-CoV-2 and H1N1 sensors at 30 min.** As incubation time increases, both SARS-CoV-2 NP and H1N1 HA plots show nonlinear response against logarithmic concentration, which are fitted by logistic model. Error bars are displayed as mean ± σ (n=3 independent experiments).

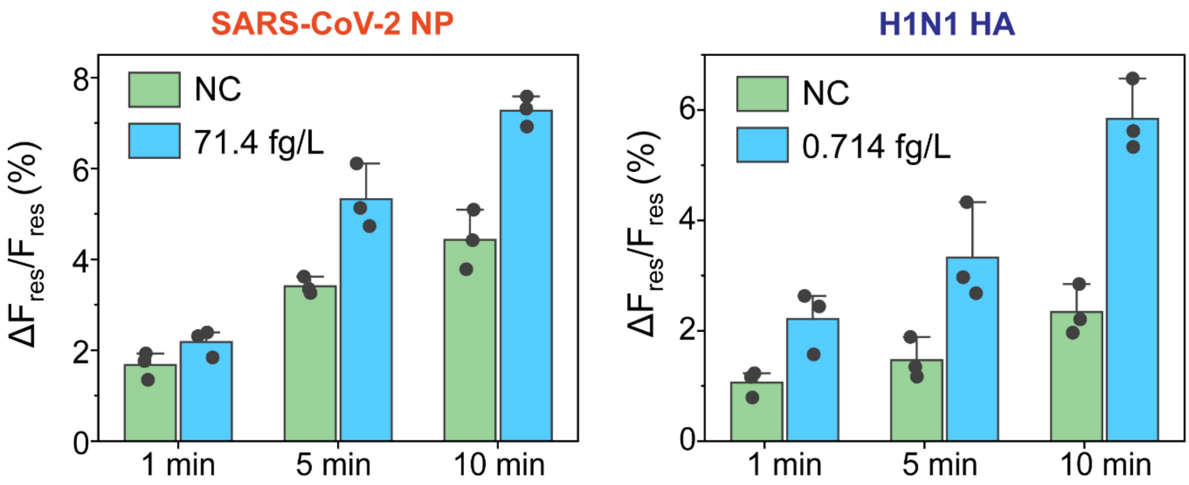


**Supplementary Fig. 23 Rapid aerosol detection capability of the SARS-CoV-2 and H1N1 sensors.** Response at 5 min is still significant enough to detect fg/L level virus antigen in aerosols, while it is more marginal for results at 1 min for low concentration virus detection. Error bars are displayed as mean ± σ (n=3 independent experiments).

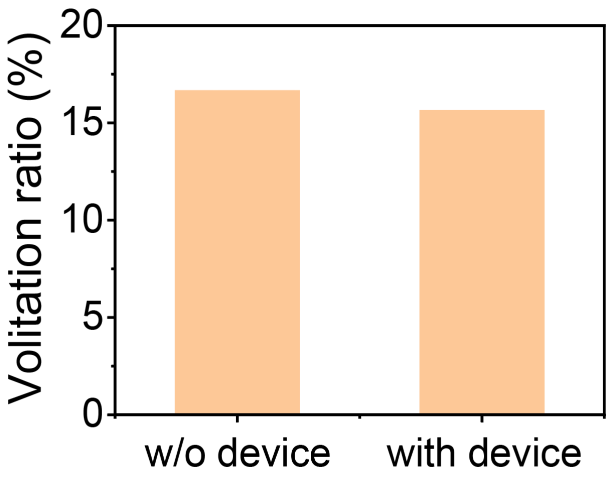


**Supplementary Fig. 24 Air permeability of face masks with and without the wireless immunoassay devices.** The volitation ratio was measured by the water vapor loss under 37°C after 6 hours with face masks capping on water-containing beakers with/without wireless immunoassay devices integrated, respectively.

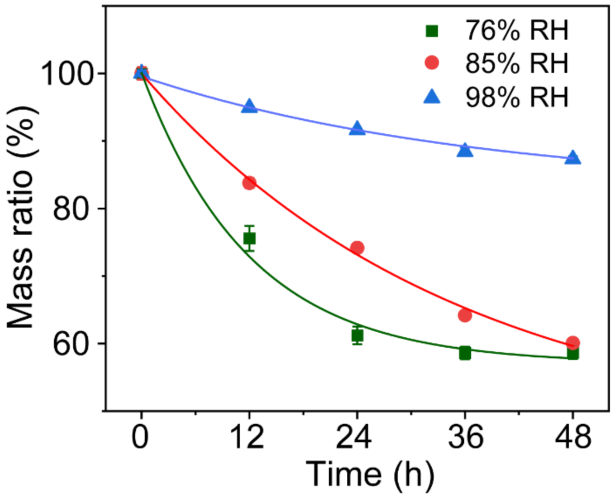


**Supplementary Fig. 25 Influence of preservation humidity on hydrogel dehydration.** The RH is modulated by storing the hydrogels in the top air of saturated saline solution ($Na_2HPO_4$, RH =98%; KCl, RH=85%, NaCl, RH=76%). Error bars are displayed as mean ± σ (n=3 independent experiments).

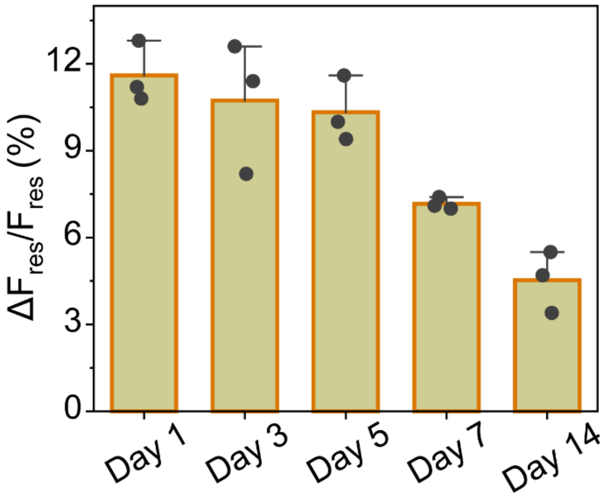


**Supplementary Fig. 26 Stability of the ImmHR sensors in two weeks.** The responses were tested with H1N1 sensors in 71.4 pg/L H1N1 antigen aerosols. The hydrogel precursors were stored in 98% RH and 4 °C for hydrogel formation. Error bars are displayed as mean ± σ (n=3 independent experiments).

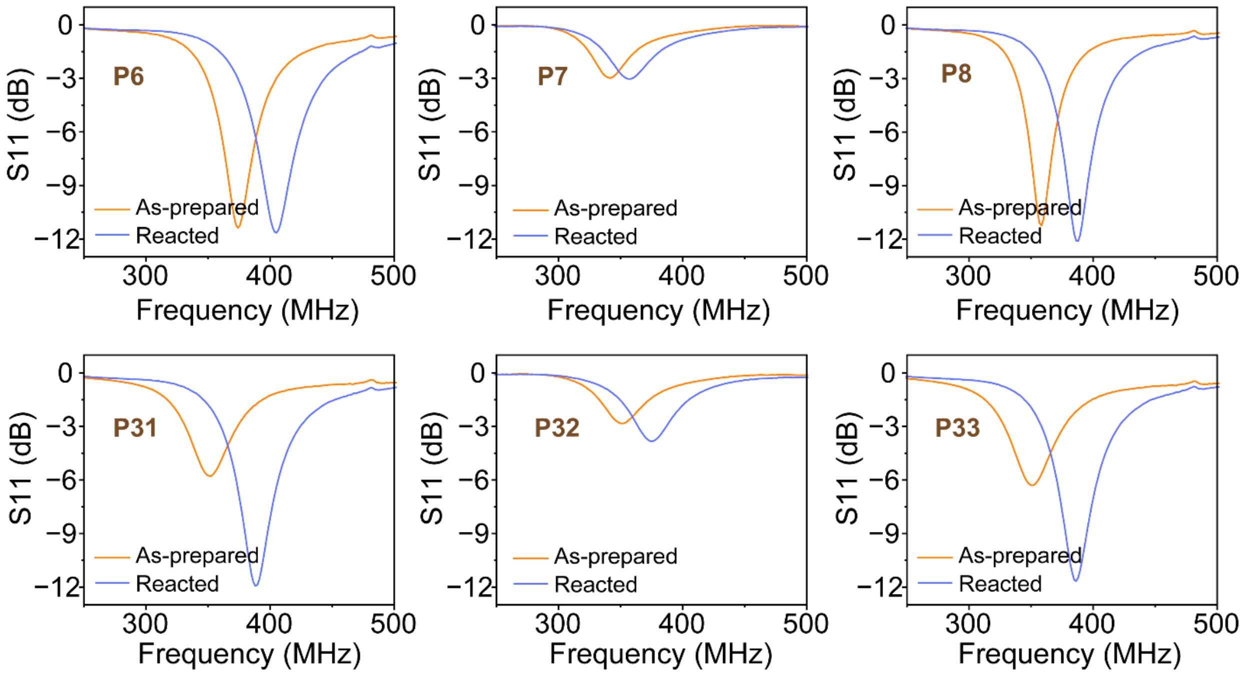


**Supplementary Fig. 27 Original response of representative clinical samples from patients with H1N1.** All the data were collected at 10 min after clinical saliva aerosol exposure.

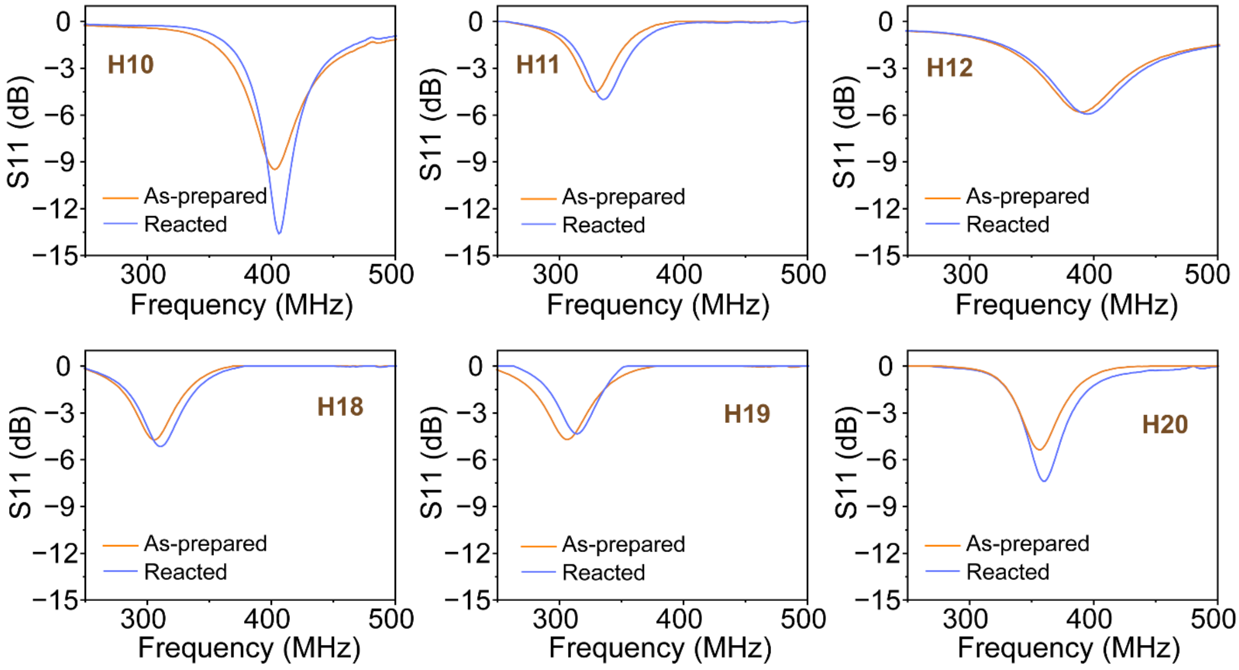


**Supplementary Fig. 28 Original response of representative samples from healthy people.** All the data were collected at 10 min after clinical saliva aerosol exposure.

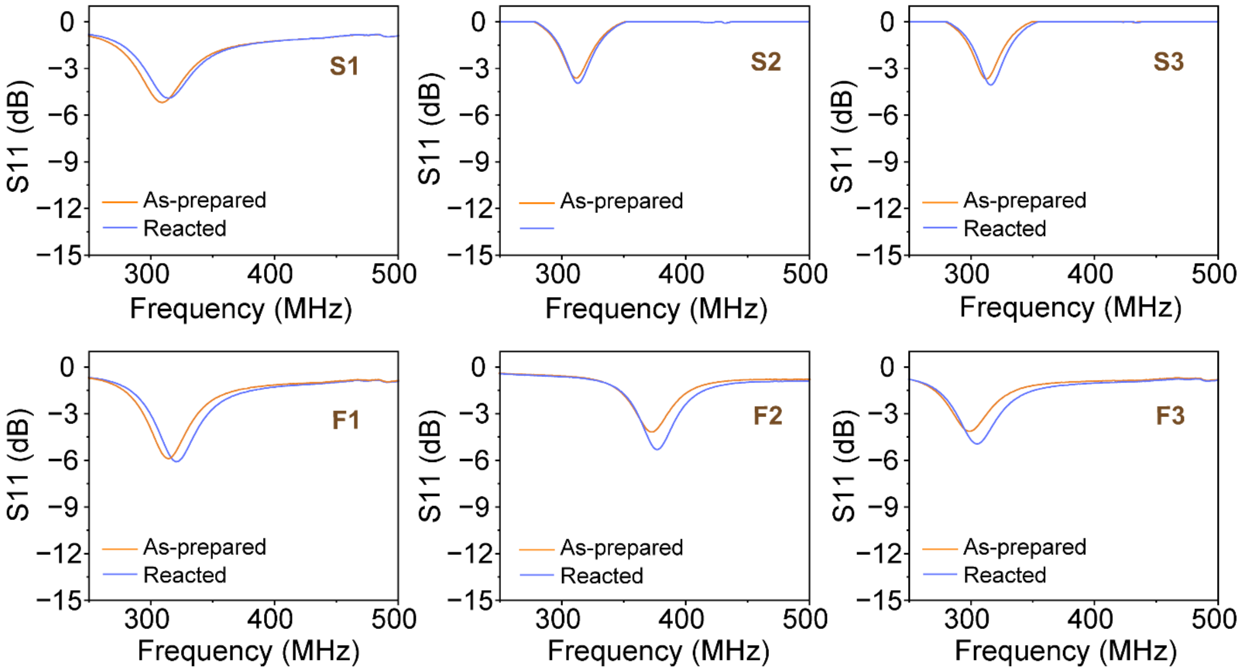


**Supplementary Fig. 29 Original response of representative clinical samples from suspected positive and fever yet H1N1 negative patients.** All the data were collected at 10 min after clinical saliva aerosol exposure.

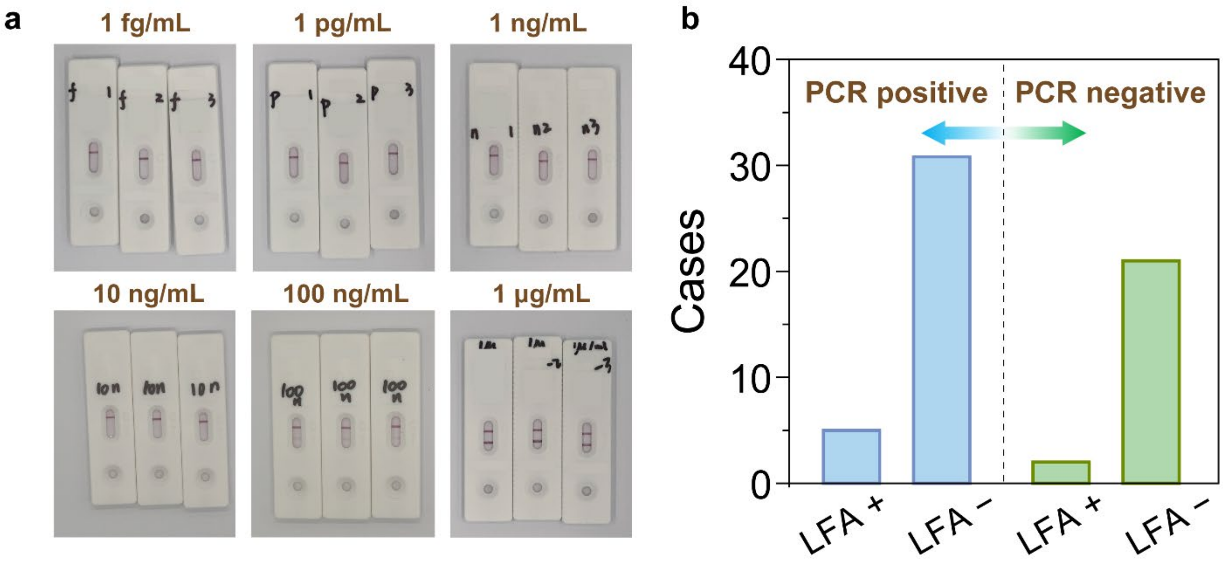


**Supplementary Fig. 30 Commercial LFA strips for H1N1 detection.**

(a) Photos of the H1N1 LFA strips in response to 1 fg/mL~1 μg/mL H1N1 HA antigen spiked in artificial saliva. (b) H1N1 LFA strips for clinical detection (n=60). The LFA strips show no response until antigen concentration increases to 100 ng/mL.

**a**

| Nucleic acid testing | | Classic: PCR | Alternative example: CRISPR |
|---|---|---|---|
| | Signal output | Fluoresence | Compatible to colorimetric, fluorecent, or electrochemical signals, etc. |
| | Devices | Benchtop PCR system | Portable or wearable devices |
| | Operations | Viral lysis, nucleic acid amplification, fluorecent detection | Viral lysis, nucleic acid amplification, detection |
| | Features | • High sensitivity, high accuracy<br>• Long time to results (1~2 h)<br>• Gold standard for clinical diagnosis | • High sensitivity ($10^{-12}$~$10^{-15}$ M)<br>• Programmability to wide working conditions<br>• Integration with other optical/electrochemical techniques for enhanced performance<br>• Complicated pretreatment, time consuming (~1 h) |

**b**

| Immunoassays | | Classic: LFA | Alternative example: ImmHR |
|---|---|---|---|
| | Signal output | Naked-eye colorimetric response | Colorimetric response and resonant response |
| | Devices | Paper-based test strips | Miniaturized resonators and portable VNA |
| | Operations | Sample collection, incubation, readout | Aerosol collection/saliva atomization, incubation, RF readout |
| | Features | • Fast response (10~15 min)<br>• Easy operation and readout<br>• Low sensitivity and accuracy | • High sensitivity (fg/L or sub-fg/L)<br>• Fast response (reliable results within 10 min and distinguishable signals as fast as at 1 min)<br>• High accuracy (AUC=0.926)<br>• Compatible to wearable or portable integration |

**Supplementary Fig. 31 Comparison of nucleic acid testing and immunoassays for virus detection.**

(a) Comparison of classic PCR and trending CRISPR methods for nucleic acid detection. (b) Comparison of classic LFA immunoassay and the wireless immunoassay reported in this work.

**Supplementary Table 1 Ct values and ImmHR sensor response of clinical samples.**

| Number | Ct value | Mark | Sensor response (%) |
|---|---|---|---|
| 1 | >40 | H1 | 0.03611 |
| 2 | >40 | H2 | 0.02352 |
| 3 | >40 | H3 | 0.01418 |
| 4 | >40 | H4 | 0.03693 |
| 5 | >40 | H5 | 0.03397 |
| 6 | >40 | H6 | 0.04339 |
| 7 | >40 | H7 | 0.04206 |
| 8 | 39.15 | S1 | 0.02217 |
| 9 | >40 | H8 | 0.02207 |
| 10 | >40 | H9 | 0.03147 |
| 11 | >40 | H10 | 0.01536 |
| 12 | 37.75 | P1 | 0.03118 |
| 13 | >40 | H11 | 0.02715 |
| 14 | >40 | H12 | 0.01193 |
| 15 | >40 | H13 | 0.01583 |
| 16 | 38.54 | S2 | 0.01662 |
| 17 | >40 | H14 | 0.02811 |
| 18 | >40 | H15 | 0.02643 |
| 19 | >40 | H16 | 0.03046 |
| 20 | 38.83 | S3 | 0.02473 |
| 21 | >40 | H17 | 0.02093 |
| 22 | >40 | H18 | 0.02267 |
| 23 | >40 | H19 | 0.04535 |
| 24 | >40 | H20 | 0.02108 |
| 25 | 21.59 | P2 | 0.10251 |
| 26 | 24.64 | P3 | 0.09475 |
| 27 | >40 | F1 | 0.02591 |
| 29 | 29.19 | P4 | 0.11563 |
| 30 | 22.68 | P5 | 0.08708 |
| 31 | 25.99 | P6 | 0.11343 |
| 32 | 27.25 | P7 | 0.0657 |
| 33 | 32.18 | P8 | 0.11424 |
| 34 | 26.36 | P9 | 0.10043 |
| 35 | >40 | F2 | 0.0232 |
| 36 | 23.2 | P10 | 0.09536 |
| 38 | 28.9 | P11 | 0.08506 |
| 39 | 20.93 | P12 | 0.11171 |
| 41 | 36.69 | P13 | 0.08201 |
| 42 | 22.32 | P14 | 0.08175 |
| 43 | 24.72 | P15 | 0.09067 |
| 44 | 28.62 | P16 | 0.1091 |
| 45 | 32.36 | P17 | 0.07673 |
| 46 | 28.25 | P18 | 0.1086 |
| 47 | 28.36 | P19 | 0.11288 |
| 48 | 31.08 | P20 | 0.10504 |
| 49 | 38.74 | S4 | 0.03159 |
| 51 | 35.84 | P21 | 0.10094 |
| 52 | 35.32 | P22 | 0.07467 |
| 53 | 25.95 | P23 | 0.10743 |
| 54 | 22.9 | P24 | 0.09678 |
| 56 | 21.91 | P25 | 0.11251 |
| 58 | 26.28 | P26 | 0.11824 |
| 59 | 34.13 | P27 | 0.09241 |
| 60 | 36.78 | P28 | 0.12001 |
| 62 | 35.7 | P29 | 0.11953 |
| 63 | 26.19 | P30 | 0.07015 |
| 64 | 27.58 | P31 | 0.12864 |
| 65 | >40 | F3 | 0.04383 |
| 66 | 24.1 | P32 | 0.12082 |
| 67 | 32.87 | P33 | 0.12937 |

Note: Participant #28, #37, #40, #50, #55, #57, #61 did not provide adequate samples for qRT-PCR tests, thus they are left out in the table.

**Supplementary Table 2 Comparison of Covid-19 detection method and this work.**

| Method | Analyte | Sample or medium | Amp | LoD | Detection time | Readout device | Ref |
|---|---|---|---|---|---|---|---|
| qRT-PCR | Viral RNA | Nasopharyngeal swabs | Yes | 11.2-21.3 copies/reaction | 1~2 h | Real-time PCR system | 4 |
| ELISA | IgG<br>IgM | Serum | No | 0.040 μg/mL (IgG)<br>0.039 μg/mL (IgM) | 1~2 h | Spectrophotometer | 5 |
| ELISA | IgG<br>IgM | Serum and plasma | No | N.A. | 3~4 h | Spectrophotometer | 6 |
| LFA | S protein | HEPES buffer | No | 5 μg/mL | 30 min | Naked eye | 7 |
| NIR-LFA | IgG<br>IgM | Serum | No | 0.236 μg/mL (IgG)<br>0.125 μg/mL (IgM) | 10 min | CMOS camera | 5 |
| NEK-LFA | IgG | Blood | Yes | 1.085 AU/mL | > 15 min | Customized imaging system | 8 |
| Fluorometric p-LFA | IgG<br>N protein | PBS | No | 185 pg/mL (S1 antibody)<br>212 pg/mL (N protein) | 20 min | Portable fluorescence scanner | 9 |
| Imaging | Virus particle | Saliva aerosol | Yes | N.A. | 30 min | Smartphone | 10 |
| AIR | S protein | Blood | No | 40.7 ng/mL | 75 min | CCD camera | 11 |
| Electrochemistry | N-gene | Nasopharyngeal swabs | No | 6.9 copies/μL | 5 min | Homemade circuit | 12 |
| Electrochemistry | S protein | Aerosol condensate | No | 10 pfu/mL | 10 min | Potentiostat | 13 |
| Electrochemistry | S protein | Antigen aerosol | No | 7 pfu/mL | 5 min | Homemade circuit | 14 |
| Electrochemistry | S protein RBD | Nasopharyngeal swabs | No | 0.73 fM | 2 h | Potentiostat | 15 |
| Electrochemistry | N protein | Spiked swab and blood | No | 1 fM | 60 min | Potentiostat | 16 |
| Electrochemistry | N protein | Nasopharyngeal swabs | No | 56 fg/mL | 10 min | EIS system | 17 |
| Electrochemistry | N protein<br>IgG<br>IgM | Saliva and blood | No | N.A. | 5~10 min | Homemade circuit | 18 |
| CRISPR | Viral RNA | Saliva | Yes | 40 copies/μL | 60 min | Microfluidic cartridge | 19 |
| CRISPR | Viral RNA | Throat swabs | Yes | N.A. | 30 min | Smartphone | 20 |
| CRISPR-LFA | Viral RNA | Virus RNA aerosol | Yes | N.A. | 90 min | Naked eye | 21 |
| CRISPR-EC | S protein<br>N protein<br>S protein RBD | Saliva and blood | Yes | 0.8 copies/μL | 2 h for RNA,<br>30 min for immunoassay | Potentiostat | 22 |
| LSPR | S protein | Nasopharyngeal swabs | No | 0.154 pg/mL | 5 min | Smartphone | 23 |
| LSPR | N-gene | PBS buffer | No | 0.18 ng/μL | 10 min | Naked eye | 24 |
| SPR | RBD | PBS | No | 1.95 nM | 10 min | Optical system | 25 |
| FET | IgG | Serum and saliva | No | 10 fM | ≤30 min | Portable meter | 26 |
| FET | S protein | PBS or nasopharyngeal swabs | No | 1 fg/mL (in PBS)<br>100 fg/mL (in CTM) | ~30 s | Semiconductor analyzer | 27 |
| FET | S protein | PBS or antigen aerosol | No | 0.1 fg/mL | 10 min | Wearable bioelectronic mask | 3 |
| FET | S protein RBD | Saliva | No | 23 fM | 10 min | Keithley Source Meter | 28 |
| MolEMS g-FET | Viral RNA | Nasopharyngeal swabs | No | 0.02 copies/μL (in VTM) | ~6.5 min | Portable system | 29 |
| Hydrogel resonator | N-gene | Nasopharyngeal swabs | Yes | 100 copies/μL | 5 h | Linear stage controller and VNA | 30 |
| ImmHR | N protein | Aerosol | No | 19.7 fg/L (10 min)<br>0.52 fg/L (30 min) | 1~30 min (10 min for assurance) | Portable VNA | This work |

Amp: amplification, N.A.: Not available, qRT-PCR: Quantitative Reverse Transcription-Polymerase Chain Reaction, ELISA: Enzyme Linked Immunosorbent Assay, LFA: Lateral Flow Assay, AIR: Arrayed Imaging Reflectometry, NIR: Near Infrared, CMOS: Complementary Metal Oxide Semiconductor, CTM: clinical transport medium, VTM: viral transport medium, NEK: nanoelectrokinetic, EIS: electrochemical impedance spectroscopy, CRISPR: Clustered Regularly Interspaced Short Palindromic Repeats, EC: Electrochemical, RBD: Receptor Binding Domain, LSPR: Localized Surface Plasmon Resonance, FET: Field Effect Transistor, MolEMS g-FET: Molecular Electromechanical System Graphene Field Effect Transistor, VNA: Vector Network Analyzer.

**Supplementary references**


1 Su, S., Yu, T., Hu, J. & Xianyu, Y. A bio-inspired plasmonic nanosensor for angiotensin-converting enzyme through peptide-mediated assembly of gold nanoparticles. *Biosens Bioelectron* **195**, 113621, (2022).

2 Dautta, M. *et al.* Programmable Multiwavelength Radio Frequency Spectrometry of Chemophysical Environments through an Adaptable Network of Flexible and Environmentally Responsive, Passive Wireless Elements. *Small Sci* **2**, 2200013, (2022).

3 Wang, B. *et al.* Wearable bioelectronic masks for wireless detection of respiratory infectious diseases by gaseous media. *Matter* **5**, 4347-4362, (2022).

4 Chan Jasper, F.-W. *et al.* Improved Molecular Diagnosis of COVID-19 by the Novel, Highly Sensitive and Specific COVID-19-RdRp/Hel Real-Time Reverse Transcription-PCR Assay Validated In Vitro and with Clinical Specimens. *J Clin Microbiol* **58**, (2020).

5 Chen, R. *et al.* Early Detection of SARS-CoV-2 Seroconversion in Humans with Aggregation-Induced Near-Infrared Emission Nanoparticle-Labeled Lateral Flow Immunoassay. *ACS Nano* **15**, 8996-9004, (2021).

6 Amanat, F. *et al.* A serological assay to detect SARS-CoV-2 seroconversion in humans. *Nat Med* **26**, 1033-1036, (2020).

7 Baker, A. N. *et al.* The SARS-COV-2 Spike Protein Binds Sialic Acids and Enables Rapid Detection in a Lateral Flow Point of Care Diagnostic Device. *ACS Cent Sci* **6**, 2046-2052, (2020).

8 Kim, C. *et al.* Nanoelectrokinetic-assisted lateral flow assay for COVID-19 antibody test. *Biosens Bioelectron* **212**, 114385, (2022).

9 Gupta, R. *et al.* Ultrasensitive lateral-flow assays via plasmonically active antibody-conjugated fluorescent nanoparticles. *Nat Biomed Eng*, (2023).

10 Kim, S. *et al.* Direct capture and smartphone quantification of airborne SARS-CoV-2 on a paper microfluidic chip. *Biosens Bioelectron* **200**, 113912, (2022).

11 Steiner, D. J. *et al.* Array-based analysis of SARS-CoV-2, other coronaviruses, and influenza antibodies in convalescent COVID-19 patients. *Biosens Bioelectron* **169**, 112643, (2020).

12 Alafeef, M., Dighe, K., Moitra, P. & Pan, D. Rapid, Ultrasensitive, and Quantitative Detection of SARS-CoV-2 Using Antisense Oligonucleotides Directed Electrochemical Biosensor Chip. *ACS Nano* **14**, 17028-17045, (2020).

13 Daniels, J. *et al.* A mask-based diagnostic platform for point-of-care screening of Covid-19. *Biosens Bioelectron* **192**, 113486, (2021).

14 Xue, Q. *et al.* An intelligent face mask integrated with high density conductive nanowire array for directly exhaled coronavirus aerosols screening. *Biosens Bioelectron* **186**, 113286, (2021).

15 Shahdeo, D. *et al.* Label free detection of SARS CoV-2 Receptor Binding Domain (RBD) protein by fabrication of gold nanorods deposited on electrochemical immunosensor (GDEI). *Biosens Bioelectron* **212**, 114406, (2022).

16 Yu, M. *et al.* An electrochemical aptasensor with N protein binding aptamer-complementary oligonucleotide as probe for ultra-sensitive detection of COVID-19. *Biosens Bioelectron* **213**, 114436, (2022).

17 Salahandish, R. *et al.* A compact, low-cost, and binary sensing (BiSense) platform for noise-free and self-validated impedimetric detection of COVID-19 infected patients. *Biosens Bioelectron* **213**, 114459, (2022).

18 Torrente-Rodriguez, R. M. *et al.* SARS-CoV-2 RapidPlex: A Graphene-Based Multiplexed Telemedicine Platform for Rapid and Low-Cost COVID-19 Diagnosis and Monitoring. *Matter* **3**, 1981-1998, (2020).
19 Chandrasekaran, S. S. *et al.* Rapid detection of SARS-CoV-2 RNA in saliva via Cas13. *Nat Biomed Eng* **6**, 944-956, (2022).
20 Zhang, T. *et al.* A paper-based assay for the colorimetric detection of SARS-CoV-2 variants at single-nucleotide resolution. *Nat Biomed Eng* **6**, 957-967, (2022).
21 Nguyen, P. Q. *et al.* Wearable materials with embedded synthetic biology sensors for biomolecule detection. *Nat Biotechnol* **39**, 1366-1374, (2021).
22 Najjar, D. *et al.* A lab-on-a-chip for the concurrent electrochemical detection of SARS-CoV-2 RNA and anti-SARS-CoV-2 antibodies in saliva and plasma. *Nat Biomed Eng* **6**, 968-978, (2022).
23 Ferreira, A. L., de Lima, L. F., Torres, M. T., de Araujo, W. R. & de la Fuente-Nunez, C. Low-Cost Optodiagnostic for Minute-Time Scale Detection of SARS-CoV-2. *ACS Nano* **15**, 17453-17462, (2021).
24 Moitra, P., Alafeef, M., Dighe, K., Frieman, M. B. & Pan, D. Selective Naked-Eye Detection of SARS-CoV-2 Mediated by N Gene Targeted Antisense Oligonucleotide Capped Plasmonic Nanoparticles. *ACS Nano* **14**, 7617-7627, (2020).
25 Akib, T. B. A. *et al.* Design and Numerical Analysis of a Graphene-Coated SPR Biosensor for Rapid Detection of the Novel Coronavirus. *Sensors (Basel)* **21**, 3491, (2021).
26 Liu, H. *et al.* Ultrafast, sensitive, and portable detection of COVID-19 IgG using flexible organic electrochemical transistors. *Sci Adv* **7**, eabg8387 (2021).
27 Seo, G. *et al.* Rapid Detection of COVID-19 Causative Virus (SARS-CoV-2) in Human Nasopharyngeal Swab Specimens Using Field-Effect Transistor-Based Biosensor. *ACS Nano* **14**, 5135-5142, (2020).
28 Guo, K. *et al.* Rapid single-molecule detection of COVID-19 and MERS antigens via nanobody-functionalized organic electrochemical transistors. *Nat Biomed Eng* **5**, 666-677, (2021).
29 Wang, L. *et al.* Rapid and ultrasensitive electromechanical detection of ions, biomolecules and SARS-CoV-2 RNA in unamplified samples. *Nat Biomed Eng* **6**, 276-285, (2022).
30 Carr, A. R. *et al.* Toward Mail-in-Sensors for SARS-CoV-2 Detection: Interfacing Gel Switch Resonators with Cell-Free Toehold Switches. *ACS Sens* **7**, 806-815, (2022).
