# Supplementary material for: Rapid and on-site wireless immunoassay of respiratory virus aerosols via hydrogel-modulated resonators
Source: Nat Commun. 2024 May 13;15:4035. doi: 10.1038/s41467-024-48294-1 (PMC11091083; doi:10.1038/s41467-024-48294-1)
Supplement: Supplementary file 3 — Reporting Summary [file 41467_2024_48294_MOESM3_ESM.pdf]

nature portfolio

Corresponding author(s): Qingjun Liu

Last updated by author(s): Mar 14, 2024

Reporting Summary

Nature Portfolio wishes to improve the reproducibility of the work that we publish. This form provides structure for consistency and transparency in reporting. For further information on Nature Portfolio policies, see our Editorial Policies and the Editorial Policy Checklist.

Statistics

For all statistical analyses, confirm that the following items are present in the figure legend, table legend, main text, or Methods section.

n/a	Confirmed	
☐	☒	The exact sample size (*n*) for each experimental group/condition, given as a discrete number and unit of measurement
☐	☒	A statement on whether measurements were taken from distinct samples or whether the same sample was measured repeatedly
☐	☒	The statistical test(s) used AND whether they are one- or two-sided<br>*Only common tests should be described solely by name; describe more complex techniques in the Methods section.*
☒	☐	A description of all covariates tested
☒	☐	A description of any assumptions or corrections, such as tests of normality and adjustment for multiple comparisons
☐	☒	A full description of the statistical parameters including central tendency (e.g. means) or other basic estimates (e.g. regression coefficient) AND variation (e.g. standard deviation) or associated estimates of uncertainty (e.g. confidence intervals)
☐	☒	For null hypothesis testing, the test statistic (e.g. *F*, *t*, *r*) with confidence intervals, effect sizes, degrees of freedom and *P* value noted<br>*Give P values as exact values whenever suitable.*
☒	☐	For Bayesian analysis, information on the choice of priors and Markov chain Monte Carlo settings
☒	☐	For hierarchical and complex designs, identification of the appropriate level for tests and full reporting of outcomes
☐	☒	Estimates of effect sizes (e.g. Cohen's *d*, Pearson's *r*), indicating how they were calculated

Our web collection on statistics for biologists contains articles on many of the points above.

Software and code

Policy information about availability of computer code

Data collection	Scanning electron microscope (GEMINI 300, Zeiss, Germany);<br>energy dispersive spectroscopy (Bruker Nano, Bruker, Germany);<br>rotational rheometer (RS6000, HAKE, US);<br>Image J.
Data analysis	Origin 2023b Learning edition;<br>CST studio Suite 2022;<br>Adobe Illustrator 2019

For manuscripts utilizing custom algorithms or software that are central to the research but not yet described in published literature, software must be made available to editors and reviewers. We strongly encourage code deposition in a community repository (e.g. GitHub). See the Nature Portfolio guidelines for submitting code & software for further information.

Data

Policy information about availability of data

All manuscripts must include a data availability statement. This statement should provide the following information, where applicable:

- Accession codes, unique identifiers, or web links for publicly available datasets
- A description of any restrictions on data availability
- For clinical datasets or third party data, please ensure that the statement adheres to our policy

All data supporting the findings described in this manuscript are available in the article and the supplementary information. Source data are provided with this paper.

Research involving human participants, their data, or biological material

Policy information about studies with human participants or human data. See also policy information about sex, gender (identity/presentation), and sexual orientation and race, ethnicity and racism.

Reporting on sex and gender	We randomly recruited 60 participants in this experiment, including 29 females and 31 males. The H1N1 positive cases include 19 females and 18 males. The H1N1 negative cases include 10 females and 13 males.
Reporting on race, ethnicity, or other socially relevant groupings	All participants are Chinese Han populations.
Population characteristics	The ages of positive cases range from 1 to 15. The ages of negative cases range from 3 to 27. The positive cases were diagnosed with respiratory infection with fever, cough, and other flu symptoms. Positive patients were taking medication to fight virus when they carried out experiments. The negative cases were healthy volunteers with no H1N1 infection and other obvious diseases during the experiments.
Recruitment	Participants were recruited randomly. All the positive participants were recruited when they arrived the hospital. All of them were confirmed as positive by PCR tests. All the negative participants were recruited by the research group through colleagues and friends. For all participants, we have no potential self-selection bias. Informed consents were received from all the participants.
Ethics oversight	Ethics Committee of Department of Biomedical Engineering, Zhejiang University ([2022]-8)

Note that full information on the approval of the study protocol must also be provided in the manuscript.

Field-specific reporting

Please select the one below that is the best fit for your research. If you are not sure, read the appropriate sections before making your selection.

☒ Life sciences ☐ Behavioural & social sciences ☐ Ecological, evolutionary & environmental sciences

For a reference copy of the document with all sections, see nature.com/documents/nr-reporting-summary-flat.pdf

Life sciences study design

All studies must disclose on these points even when the disclosure is negative.

Sample size

[1] 60 volunteers have been included in this experiment.
[2] No sample-size calculation is performed. The sample-size is chosen by referring to other literatures using biosensors for virus detection.

References

1. Chandrasekaran, S. S. et al. Rapid detection of SARS-CoV-2 RNA in saliva via Cas13. Nat Biomed Eng 6, 944-956, (2022).
2.Wang, L. et al. Rapid and ultrasensitive electromechanical detection of ions, biomolecules and SARS-CoV-2 RNA in unamplified samples. Nat Biomed Eng 6, 276-285, (2022).
3. Zhang, T. et al. A paper-based assay for the colorimetric detection of SARS-CoV-2 variants at single-nucleotide resolution. Nat Biomed Eng 6, 957-967, (2022).
4.Najjar, D. et al. A lab-on-a-chip for the concurrent electrochemical detection of SARS-CoV-2 RNA and anti-SARS-CoV-2 antibodies in saliva and plasma. Nat Biomed Eng 6, 968-978, (2022).
5.Guo, K. et al. Rapid single-molecule detection of COVID-19 and MERS antigens via nanobody-functionalized organic electrochemical transistors. Nat Biomed Eng 5, 666-677, (2021).
6.Gupta, R. et al. Ultrasensitive lateral-flow assays via plasmonically active antibody-conjugated fluorescent nanoparticles. Nat Biomed Eng, (2023).
7.Nguyen, P. Q. et al. Wearable materials with embedded synthetic biology sensors for biomolecule detection. Nat Biotechnol 39, 1366-1374, (2021).
8.Daniels, J. et al. A mask-based diagnostic platform for point-of-care screening of COVID-19. Biosens Bioelectron 192, 113486, (2021).
9.Xue, Q. et al. An intelligent face mask integrated with high density conductive nanowire array for directly exhaled coronavirus aerosols screening. Biosens Bioelectron 186, 113286, (2021).

Data exclusions	No data point excluded.
Replication	All experiments except clinical tests were replicated at least three times. Clinical tests were replicated at once.
Randomization	For positive cases, the participants were recruited randomly when they arrived the hospital. For negative cases, the participants were recruited before confirming their health status using rapid detection kit.
Blinding	Blinding is not relevant to this study because the aim of this work is to verify the sensor with standard testing techniques such as PCR, so negative and positive groups has to be clear for evaluating the sensitivity and specificity of the sensing devices proposed in this work.

Reporting for specific materials, systems and methods

We require information from authors about some types of materials, experimental systems and methods used in many studies. Here, indicate whether each material, system or method listed is relevant to your study. If you are not sure if a list item applies to your research, read the appropriate section before selecting a response.

Materials & experimental systems

n/a	Involved in the study	
☐	☒	Antibodies
☒	☐	Eukaryotic cell lines
☒	☐	Palaeontology and archaeology
☒	☐	Animals and other organisms
☒	☐	Clinical data
☒	☐	Dual use research of concern
☒	☐	Plants

Methods

n/a	Involved in the study	
☒	☐	ChIP-seq
☒	☐	Flow cytometry
☒	☐	MRI-based neuroimaging

Antibodies

Antibodies used	Antibodies were purchased from Sino Biologicals. SARS-CoV-2 NP antibody (rabbit mAb, 40143-R001), H1N1 HA antibody (rabbit mAb, 11684-MM05), and RSV FP antibody (rabbit mAb, 11049-R338).
Validation	https://www.sinobiological.com/antibodies/cov-nucleocapsid-40143-r001<br>https://www.sinobiological.com/antibodies/hemagglutinin-ha-11684-mm05<br>https://www.sinobiological.com/antibodies/rsv-fusion-11049-r338

Plants

Seed stocks	Not relevant.
Novel plant genotypes	Not relevant.
Authentication	Not relevant.
